# Resistance against *Sclerotinia sclerotiorum* in soybean involves a reprogramming of the phenylpropanoid pathway and up‐regulation of antifungal activity targeting ergosterol biosynthesis

**DOI:** 10.1111/pbi.13082

**Published:** 2019-02-11

**Authors:** Ashish Ranjan, Nathaniel M. Westrick, Sachin Jain, Jeff S. Piotrowski, Manish Ranjan, Ryan Kessens, Logan Stiegman, Craig R. Grau, Shawn P. Conley, Damon L. Smith, Mehdi Kabbage

**Affiliations:** ^1^ Department of Plant Pathology University of Wisconsin‐Madison Madison WI USA; ^2^ The Great Lakes Bioenergy Research Center University of Wisconsin‐Madison Madison WI USA; ^3^ School of Computational and Integrative Sciences Jawaharlal Nehru University New Delhi India; ^4^ Department of Agronomy University of Wisconsin‐Madison Madison WI USA; ^5^ Present address: Yumanity Therapeutics Cambridge MA USA

**Keywords:** necrotrophic fungi, resistance, *Sclerotinia sclerotiorum*, antifungal metabolites, ergosterol, reactive oxygen species, jasmonate signalling, phenylpropanoid pathway

## Abstract

*Sclerotinia sclerotiorum*, a predominately necrotrophic fungal pathogen with a broad host range, causes a significant yield‐limiting disease of soybean called Sclerotinia stem rot. Resistance mechanisms against this pathogen in soybean are poorly understood, thus hindering the commercial deployment of resistant varieties. We used a multiomic approach utilizing RNA‐sequencing, gas chromatography–mass spectrometry‐based metabolomics and chemical genomics in yeast to decipher the molecular mechanisms governing resistance to *S. sclerotiorum* in soybean. Transcripts and metabolites of two soybean recombinant inbred lines, one resistant and one susceptible to *S. sclerotiorum* were analysed in a time course experiment. The combined results show that resistance to *S. sclerotiorum* in soybean is associated in part with an early accumulation of JA‐Ile ((+)‐7‐iso‐jasmonoyl‐L‐isoleucine), a bioactive jasmonate, increased ability to scavenge reactive oxygen species, and importantly, a reprogramming of the phenylpropanoid pathway leading to increased antifungal activities. Indeed, we noted that phenylpropanoid pathway intermediates, such as 4‐hydroxybenzoate, cinnamic acid, ferulic acid and caffeic acid, were highly accumulated in the resistant line. *In vitro* assays show that these metabolites and total stem extracts from the resistant line clearly affect *S. sclerotiorum* growth and development. Using chemical genomics in yeast, we further show that this antifungal activity targets ergosterol biosynthesis in the fungus, by disrupting enzymes involved in lipid and sterol biosynthesis. Overall, our results are consistent with a model where resistance to *S. sclerotiorum* in soybean coincides with an early recognition of the pathogen, leading to the modulation of the redox capacity of the host and the production of antifungal metabolites.

## Introduction

*Sclerotinia sclerotiorum* (Lib.) de Bary is a plant fungal pathogen with a predominately necrotrophic lifestyle and worldwide distribution that is known to infect over 400 plant species (Boland and Hall, 1994). On soybean (*Glycine max* (L.) Merr.), it causes sclerotinia stem rot (SSR), a significant and challenging yield‐limiting disease. SSR development is heavily influenced by weather conditions, and disease development is favoured by cool and wet conditions during flowering. Data suggest that 1.6 billion kilograms of soybean is lost each year to SSR in the US alone, making it the second most damaging disease of soybean (Baker *et al*., 2010. Globally, SSR can cause yield reductions as high as 60% (Cunha *et al*., 2010). Soybeans are an important source of plant proteins and oils globally (Hartman *et al*., 2011; Hill *et al*., 2006; Ribeiro *et al*., 2017); these products can also be significantly affected by SSR (Hoffman *et al*., 1998). Management strategies against SSR rely largely on chemical control (McCaghey *et al*., 2017), though cultural practices such as crop rotation, seeding rate and row spacing modifications have been used with limited success. While genetic resistance is by far more sustainable, our understanding of resistance mechanisms against this pathogen is limited, and current commercial varieties lack adequate levels of resistance to SSR.

In the past two decades, studies have increased our understanding of *S. sclerotiorum* pathogenic development*. S. sclerotiorum* is a prolific producer of cell wall degrading enzymes (CWDEs) that contribute to its pathogenic success (Amselem *et al*., 2011). In addition to its lytic repertoire, the pathogenic success of *S. sclerotiorum* relies on the key virulence factor oxalic acid (OA). Mutants that are defective in OA production are weakly pathogenic (Kabbage *et al*., 2013; Liang *et al*., 2015; Williams *et al*., 2011; Xu *et al*., 2015). In the addition to OA, other virulence factors targeting host responses are proposed to contribute to the pathogenicity of *S. sclerotiorum*; Pedras and Ahiahonu, 2004; Zhu *et al*., 2013). Unfortunately, these studies have largely focused on the fungal side of this interaction, and only provide glimpses into the plant mechanisms governing resistance to this pathogen, mainly in model plants. In soybean, bi‐parental linkage mapping led to the discovery of many quantitative trait loci (QTL) for resistance to this pathogen. Remarkably, a total of 103 QTL on 18 of the 20 soybean chromosomes have been recorded in SoyBase (Grant *et al*., 2009) with minimal overlap between QTL reported by different studies (Arahana *et al*., 2001; Huynh *et al*., 2010; Kim and Diers, 2000; McCaghey *et al*., 2017; Moellers *et al*., 2017; Wei *et al*., 2017). Despite these efforts, gene level and mechanistic details of soybean resistance mechanisms against *S. sclerotiorum* remain unknown.

Recent advances in Next‐Generation RNA sequencing (RNAseq) allow for cost‐efficient and powerful examination of global differences in the transcriptional response to environmental cues. The application of RNAseq approaches in soybean–*S. sclerotiorum* interaction studies will, most assuredly, contribute to the development of molecular genetic resources crucial for mechanistic and translational research. Transcriptomics were used to study the interaction of *S. sclerotiorum* with non‐model plant hosts, including soybean (Calla *et al*., 2009, 2014), canola (Girard *et al*., 2017; Joshi *et al*., 2016; Seifbarghi *et al*., 2017), pea (Zhuang *et al*., 2012) and common bean (Oliveira *et al*., 2015). While informative, these studies were solely based on gene expression comparisons, and thus may not provide a complete picture of the flow of biologic information. When coupled with other omics approaches, such as metabolomics and chemical genomics, and functional studies, RNAseq can provide a much greater understanding of the biology, by integrating genetic responses of a particular interaction to functional consequences. Herein, we apply multiomics approaches to identify and validate resistance processes against *S. scleoriorum* in soybean lines generated in our breeding programme. The identification of these processes will not only increase our understanding of the soybean–*S. sclerotiorum* interaction but will also facilitate the introgression of resistance into soybean varieties.

Our recent breeding efforts led to the identification of several recombinant inbred lines (RILs) highly resistant to *S. sclerotiorum* (McCaghey *et al*., 2017), using the soybean line W04‐1002 as the main source of resistance (Peltier *et al*., 2009). After multiple generations of greenhouse selection, we have chosen two RILs originating from the same cross: one resistant (91‐145) and one susceptible (91‐44) to SSR to complete this study. A comparative analysis using a combination of RNAseq, metabolomics and chemical genomics in yeast shows that resistance in 91–145 is associated in part with an earlier induction of jasmonate signalling, increased ability to scavenge reactive oxygen species (ROS) and importantly a reprogramming of the phenylpropanoid pathway leading to increased antifungal activities. We further discuss and provide evidence for the importance of antifungal compounds during the resistance response against this pathogen and show that this antifungal activity targets ergosterol biosynthesis in the fungus. We propose that the modulation of the identified pathways through RNAi/gene editing or overexpression approaches may be used to introgress SSR resistance in commercial soybean germplasm and possibly other host crops.

## Results

### Disease development in the resistant (91‐145) and susceptible (91‐44) soybean lines

**Figure 1:**
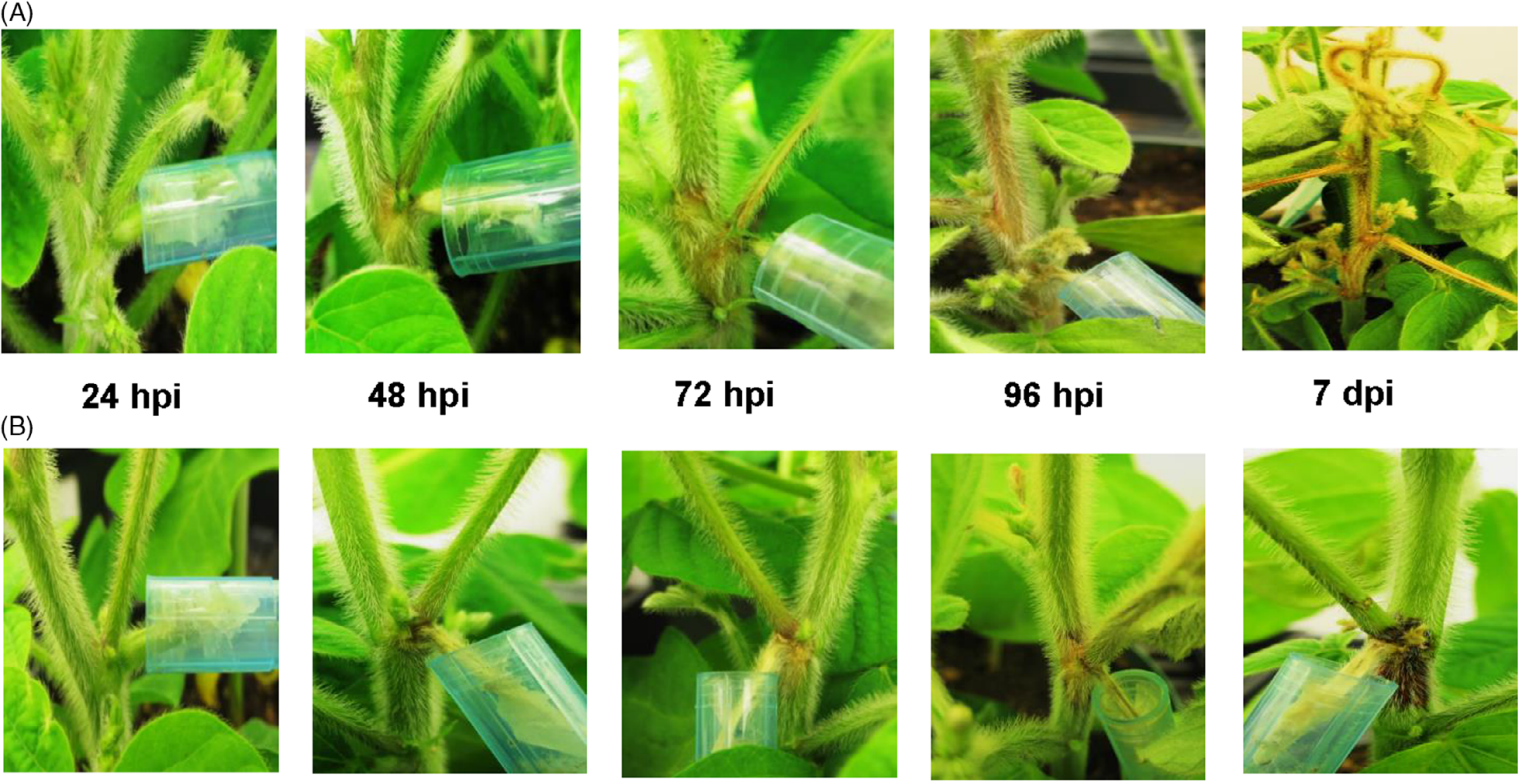
Symptom development in susceptible and resistant soybeans. Disease symptoms observed following petiole inoculation with an agar plug containing actively growing mycelia of *S. sclerotiorum* at 24, 48, 72, 96 h post‐inoculation (hpi) and 7 days post‐inoculation (dpi). (A) Susceptible (S) line. (B) Resistant (R) line. At 7 dpi in the R line, red coloration at point of inoculation (red node) is prominently visible.

Two recombinant inbred lines (RIL) of soybean showing a differential response to *S. sclerotiorum* were chosen for this study. The resistant and susceptible selections were classified based on our previous germplasm selection study (McCaghey *et al*., 2017). Both the resistant 91‐145 (R) and the susceptible 91‐44 (S) lines were developed utilizing the SSR resistant parental line W04‐1002 (P1) and LN89‐5717 (PI 5745542), an SSR‐susceptible parental line having other desirable pathogen resistance traits (McCaghey *et al*., 2017). Plants were inoculated using the cut petiole inoculation method (Ranjan *et al*., 2018) and infection progression was monitored in R and S soybean lines over 7 days. Initial symptoms of SSR began appearing on the main stem 48 h post‐inoculation (hpi) as light brown lesions surrounding the point of inoculation, which spread as the disease progressed (Figure 1). By 96 hpi, extensive tissue colonization occurred along the main stem in the S line, while small restricted lesions with a red coloration were observed at the inoculation site in the R line (Figure 1). By Day 7, it was apparent that the R line had largely restricted fungal growth on the main stem and the red coloration observed at the site of infection had become more prominent, whereas the *S. sclereotiorum* infection had girdled the main stem of the S line (Figure 1). Global transcriptome and metabolome analyses were conducted on *S. sclereotiorum‐*infected soybean stem tissue collected from both R and S lines at the specified time points.

### Mapping and overview of RNA sequencing data

RNA sequencing was conducted on 24 stem samples, consisting of three independent biological replicates for each of the time points selected. Depending on the time point, 64.9–89.7 million raw reads were generated, with 95.7%–96.6% of reads mapping to the reference genomes of soybean and *S. sclereotiorum* at all time points. On average, 96% of the total reads mapped uniquely to the soybean reference genome in the uninfected plants of both lines. In the S line, 91.6%, 91.8% and 68.9% of the reads mapped to the soybean genome at 24, 48 and 96 hpi respectively. In the R line, 92.8%, 91.9% and 88.2% of the reads mapped to the soybean genome at 24, 48 and 96 hpi respectively (Table 1). At 96 hpi, the reads mapping to the fungal genome in the S line (27%) is significantly higher than those in R line (8%), which is expected considering the extensive colonization of the soybean stem by *S. sclerotiorum* in the susceptible interaction, particularly at the later stages of the infection process.

**Table 1:** Summary of the sequencing metrics of the RNA‐seq

Time points (hours)	Total reads	Mapping to *Glycine Max*	Mapping to *S. sclerotiorum*
Susceptible line (S)			
Control	79,559,096	76,737,931 (96.5%)	0 (0.0%)
24 hpi	81,259,978	74,457,988 (91.6%)	3,607,921 (4.4%)
48 hpi	74,344,340	68,265,965 (91.8%)	2,941,195 (4.0%)
96 hpi	69,266,099	47,765,044 (68.9%)	18,712,374 (27.0%)
Resistant line (R)			
Control	89,753,272	85,133,076 (96.6%)	0 (0.0%)
24 hpi	64,923,300	60,261,793 (92.8%)	2,286,347 (3.5%)
48 hpi	67,520,185	62,099,244 (91.9%)	2,513,457 (3.8%)
96 hpi	72,951,207	64,399,890 (88.2%)	5 691,551 (7.8%)

### Differentially expressed genes (DEGs) of soybean during infection

A comparative analysis of differentially expressed genes (DEGs) was performed during the course of infection in both the R and S lines as well as between these two lines at the specified time points. In the R line, we observed the maximum number of DEGs at 48 h following inoculation (Table S1 and Figure S1A). In contrast, the maximum DEGs in the S line occurred 96 h post‐inoculation (Table S1 and Figure S1B). Interestingly, at 96 hpi, the number of DEGs in the S line (14 050) was markedly higher than the R line (2442), suggesting that during the resistant response, a return to homoeostasis is observed at the later stages of infection. In total, 16 462 unique DEGs were identified in our lines during the course of infection (Figure S1C). Among these, 7319 were differentially regulated in both R and S lines, while 920 were strictly associated with the R line and 8223 were S line specific (Figure S1C). These results indicate that *S. sclerotiorum* infection causes a dramatic change in gene expression in soybean (∼19% of total annotated genes in *Glycine max* genome) and suggests substantial differences between the resistant and susceptible soybean lines used in this study in response to *S. sclerotiorum* challenge.

Pairwise comparisons of DEGs were performed between R and S soybean lines at the selected time points (Table S2 and Figure S1D). At 24 and 48 hpi, 1039 and 803 DEGs were identified between the two lines respectively. However, the number of DEGs sharply increased to 2087 at 96 hpi. This is consistent with the distinct patterns of gene expression in the S and R lines at the later stages of infection (Figure S1A,B).

### Gene ontology enrichment and biological process analyses

**Figure 2:**
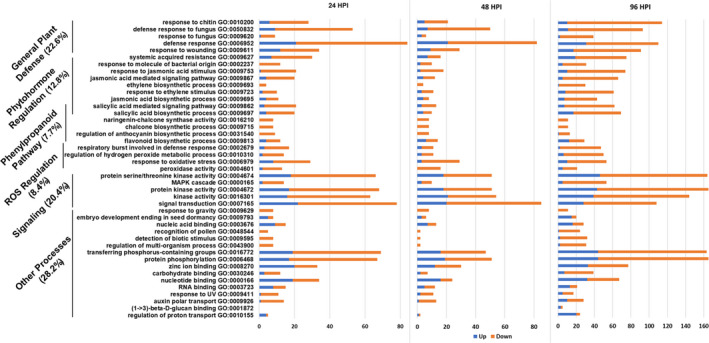
Significantly enriched gene ontology (GO) biological processes in R line compared to S line at different time points following infection with *S. Sclerotiorum*. The *y*‐axis represents significantly enriched GO processes which were enriched (FDR < 0.05) at at least one time point of infection (24, 48 and 96 hpi). The *x*‐axis indicates the total number of genes annotated to each GO process. Orange sections represent down‐regulated genes while blue sections represent up‐regulated genes.

We focus the reminder of this manuscript on direct comparisons between resistant and susceptible soybean lines, to single out potential processes associated with resistance to *S. sclerotiorum* in soybean. Soybean gene locus IDs identified through the DEG analysis were used to perform gene ontology (GO) enrichment analysis using the Soybase gene model data mining and analysis tool (Morales *et al*., 2013). A false discovery rate (FDR) value of 0.05 was used to identify significantly regulated GO biological processes, and individual GO processes were considered in this analysis if they were significantly enriched in at least one of the time points used (Table S3 and Figure 2). Our data show an overrepresentation of genes in GO terms related to signal transduction (i.e. kinase signalling, phosphorylation), plant defence responses (i.e. response to chitin, fungi), phenylpropanoid pathway (i.e. chalcones, anthocyanins, flavonoids, salicylic acid), ROS scavenging and the biosynthesis/regulation of phytohormones (i.e. jasmonic acid, salicylic acid, ethylene). DEGs underrepresented throughout the course of infection include gene related to nucleotide binding and zinc ion binding. While the involvement of categories such as ROS, defence responses and phytohormone regulation is not surprising, our results point to complex mechanisms of gene regulation associated with resistance to *S. sclerotiorum*. The differential regulation of phenylpropanoid pathway genes is intriguing and will be the subject of further characterization below.

### Metabolite profiling of susceptible and resistant soybean lines in response to *S. sclerotiorum*

Transcriptomic analysis was complemented by metabolite profiling of stem samples collected from our resistant and susceptible soybean lines following *S. sclerotiorum* infection. Simple changes in transcript levels might not always correlate with biological activity, but changes in metabolite flux are quantifiable outcomes that can directly explain disease phenotypes. Gas chromatography‐mass spectrometry (GC‐MS) analysis was performed to broadly evaluate metabolite profiles in control and infected soybean stems at 48 and 72 hpi. Three independent biological replicates were used for each time point. A total of 360 metabolites were detected, but only 164 could be identified based on available databases (Table S4). Each metabolite was characterized by its distinct retention time and mass to charge ratio (m/z). All the 164 identified metabolites found in infected soybean were also detected in non‐inoculated stems and therefore are likely of plant origin. Despite this, we note that as disease progresses, contributions from *S. sclerotiorum* cannot be ruled out.

**Figure 3:**
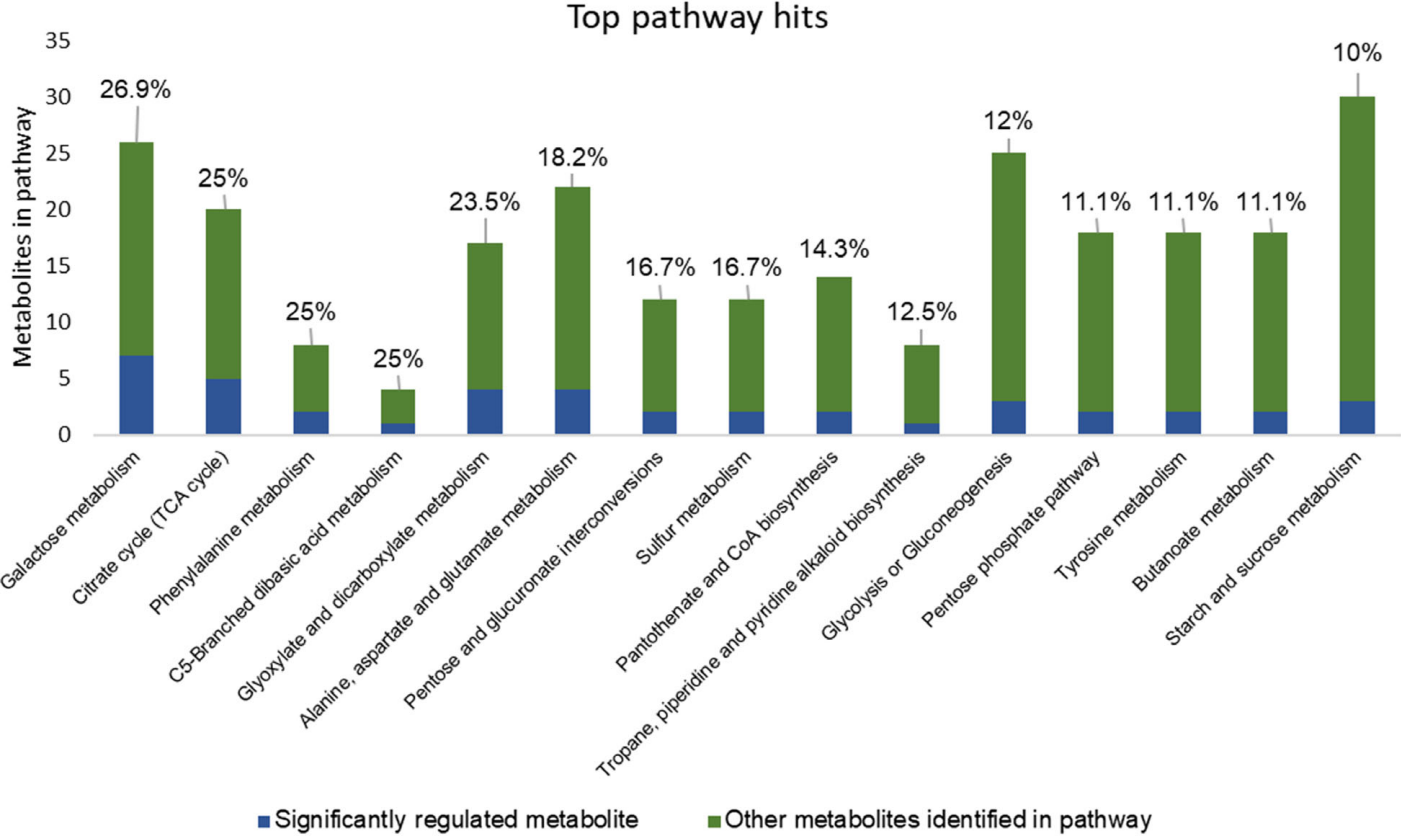
Pathway analysis of metabolites differentially detected between the R and S lines at 48 and/or 72 hpi. The *y*‐axis represents the annotated metabolites in each pathway. Blue sections represent the differentially detected metabolites, whereas green sections represent the remaining annotated metabolites in the pathway. Percentages denote the portion of the pathway found to be differentially detected. Only pathways with at least 10% of their annotated metabolites showing significant regulation were included.

**Figure 4:**
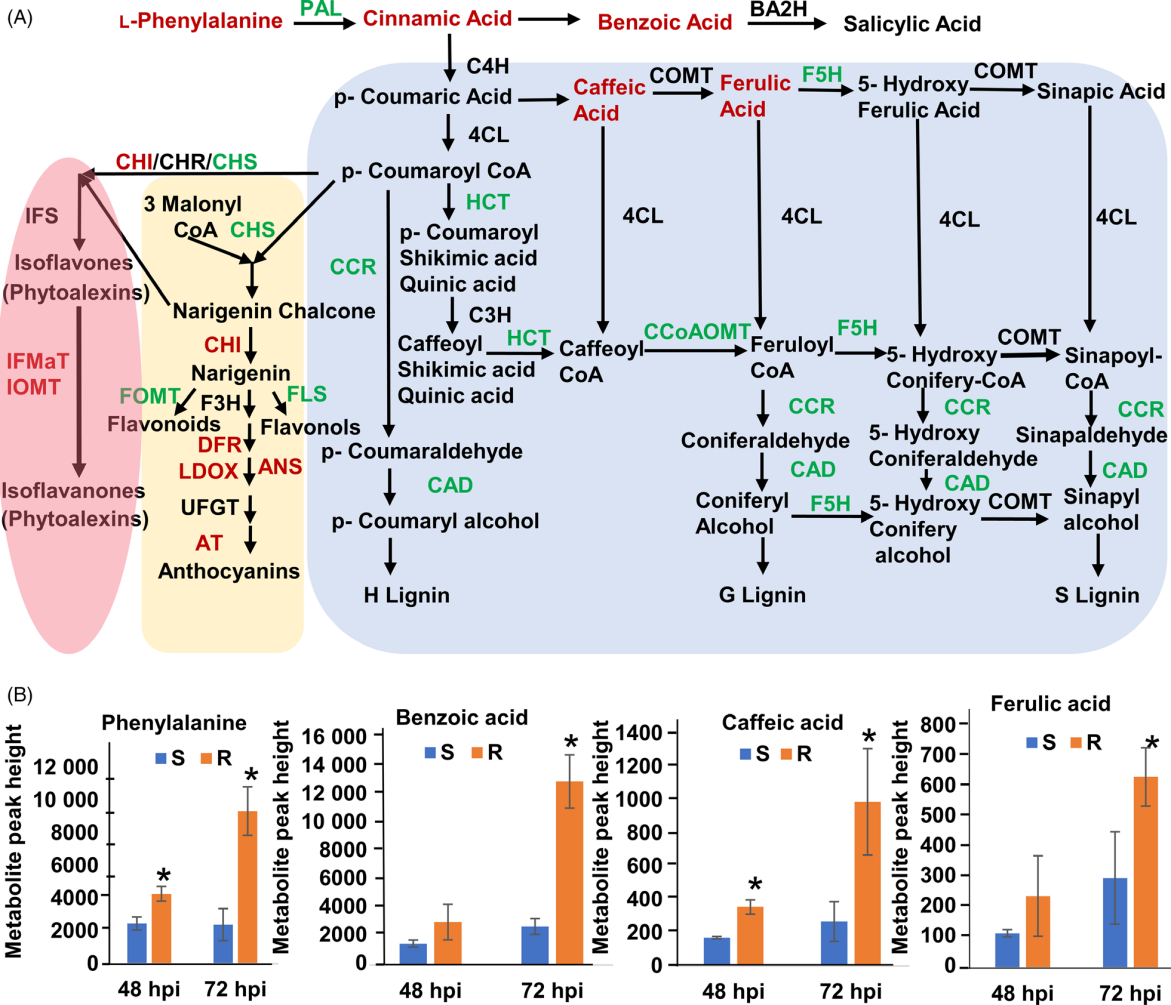
DEGs and metabolites involved in the phenylpropanoid pathway in R and S soybean lines following *S. sclerotiorum* infection. (A) Enzymes are indicated in uppercase letters. Gene names indicated in red or green represent significantly up‐regulated or down‐regulated genes in the R line compared to the S line respectively. The metabolite name in red indicates significantly up‐regulated phenylpropanoid pathway intermediates in the R line compared to the S line. PAL (phenylalanine ammonia‐lyase); BA2H (benzoic acid 2‐hydroxylase); C4H (cinnamate 4‐hydroxylase); COMT (caffeic O‐methyltransferase); F5H (ferulic acid 5‐hydroxylase); 4CL (4‐coumarate: CoA ligase); C3H (p‐coumarate 3 hydroxylase); HCT (N‐hydroxycinnamoyl transferase); CCoAOMT (caffeoyl‐CoA O‐methyltransferase); F5H (flavanone 5‐hydroxylase); CCR (cinnamoyl‐CoA reductase); CAD (cinnamoyl alcohol dehydrogenase); CHS (chalcone synthase); CHI (chalcone isomerase); CHR (chalcone reductase); FOMT (Flavonoid 4′‐O‐methyltransferase); FLS (Flavonol synthase); F3H (flavanone 3‐hydroxylase); DHFR (dihydroflavonol‐4‐ reductase); ANS (anthocyanidin synthase also called LDOX, leucoanthocyanidin dioxygenase); ANS (anthocyanidin synthase); UFGT (UDP‐flavonoid glucosyltransferase); AT (anthocyanin acyltransferase); IFS (isoflavone synthase); IFMaT (isoflavone 7‐O‐glucoside‐6′’‐O‐malonyltransferase); IOMT (isoflavone methyltransferase); this figure was adapted from Baxter and Neal Stewart, 2013 and Ferrer *et al*., 2008 (B) Estimation of the phenylpropanoid pathway intermediate metabolites (peak height) phenylalanine, benzoic acid, 3, 4‐dihydroxycinnamic acid (caffeic acid) and ferulic acid in R and S line at 48 and 72 hpi using GC–MS. Data are presented as means ± standard deviation (SD) from three independent biologically replicate with each replicate containing four pooled stem samples. *Indicates a significant difference at *P*‐value <0.05 (t‐test).

MetaboAnalyst 3.0 (Xia *et al*., 2015) was used for the analysis of the 164 identified metabolites. One‐way ANOVA using Fisher's least significant difference method (Fisher's LSD) identified 80 metabolites, the accumulation of which was significantly (FDR < 0.05) affected by *S. sclerotiorum* inoculation at 48 and 72 hpi in R line compared to S line (Table S5). These metabolites included polar compounds, such as nucleotides, amino acids, alcohols, organic acids and carbohydrates, along with non‐polar compounds including fatty acids, and long‐chain alcohols (Table S5). The multivariate analysis of identified metabolites was performed using Partial Least Squares‐Discriminant Analysis (PLS‐DA). The analysis showed distinct metabolomic profiles at each time point during the course of infection, culminating with the largest segregation of metabolites between the R and S lines at 72 hpi (Figure S2). The fold change of significantly regulated metabolites during this time course is indicated in Table S5. Significantly regulated metabolites were assigned to distinct functional categories according to the chemical groups to which they belong (Table S5) and to specific plant pathways in which they may function (Table S6). Our data revealed several differentially regulated metabolic processes between the R and S lines (Figure 3). However, those involved in phenylalanine metabolism are particularly interesting given the differential expression of phenylpropanoid pathway genes identified in transcriptomic analysis (Figure 4A) and the important role of this pathway in plant defence. Indeed, the precursor of the phenylpropanoid pathway, phenylalanine and downstream intermediate metabolites, such as benzoic acid, caffeic acid and ferulic acid, is highly accumulated in the stem of the R line compared to the S line following *S. sclerotiorum* infection (Figure 4B). Overall, our transcriptomics and metabolomics data suggest a key participation of the phenylpropanoid pathway in resistance to *Sclerotinia sclerotiorum*.

Similar to transcriptomic data, the comparative metabolite profiles also implicated phytohormones in this interaction. Namely, the fatty acids linolenic acid (a precursor of jasmonic acid) and cyanoalanine (an indicator of ethylene biosynthesis; Wasternack and Song, 2017; Yip and Yang, 1998) are both significantly induced at 48 and 72 hpi, in the R line (Figure S3). Interestingly, the most highly up‐regulated metabolite in our R line is mucic acid with an ~86‐fold higher accumulation (Table S5). Mucic acid, also referred to as galactaric acid, can be produced by the oxidation of d‐galacturonic acid, the main component of pectin. Galactose metabolism and the TCA cycle were the pathways most affected by this analysis, and the metabolites assigned to them were primarily carbohydrates and organic acids respectively. Interestingly, of the metabolites down‐regulated in R plants in comparison to S plants, 81.8% (9/11) belonged to one of these two groups (Table S5). Although these metabolites relate to multiple pathways, their down‐regulation in R plants may be a strategy to reduce *S. sclerotiorum* access to preferable carbon sources.

### Reprogramming of the phenylpropanoid pathway in resistance to *Sclerotinia sclerotiorum*

Many secondary metabolites derived from multiple branches of the phenylpropanoid pathway, including lignin, isoflavonoid phytoalexins and other phenolic compounds such as benzoic acid, have been proposed as important components of defence responses (Naoumkina *et al*., 2010; Piasecka *et al*., 2015). In this study, we noted the differential regulation of transcripts and metabolites related to the phenylpropanoid pathway between the R and S soybean lines. At the transcript level, we observed a down‐regulation of genes encoding phenylalanine ammonia‐lyase (PAL), lignin biosynthetic enzymes, ferulate 5‐hydroxylase (F5H), N‐hydroxycinnamoyl/benzoyltransferase (HCT), caffeoyl‐CoA O‐methyltransferase (CCoAOMT), cinnamoyl‐CoA reductase (CCR), cinnamyl alcohol dehydrogenase (CAD), chalcone synthase (CHS), flavonol synthase (FLS) and flavonoid 4′‐O‐methyltransferase (FOMT) in R line compared to S line (Table 2 and Figure 4A). Coincidentally, genes coding for enzymes involved in anthocyanin and phytoalexin biosynthesis were up‐regulated in the R line, these include, anthocyanidin synthase (ANS), anthocyanin acyltransferase (AT), dihydroflavonol reductase (DFR), isoflavone 7‐O‐glucoside‐6′’‐O‐malonyltransferase (IFMaT) isoflavone 7‐O‐methyltransferase (IOMT) and isoflavone reductase (IFR) (Table 2 and Figure 4A). Transcript levels of select genes within these pathways were validated using RT‐qPCR, thus confirming the RNA‐Seq results (Figure S4).

**Table 2:** A list of differentially expressed genes (DEGs) within the soybean phenylpropanoid pathway

Gene locus	24 hpi^a^	48 hpi^a^	96 hpi^a^	Description
Glyma.03G181700	–	–	−1.08	Phenylalanine ammonia‐lyase (PAL)
Glyma.03G181600	–	−0.60	−1.20	Phenylalanine ammonia‐lyase (PAL)
Glyma.02G309300	–	–	−1.28	Phenylalanine ammonia‐lyase (PAL)
Glyma.09G186400	–	–	−1.05	Ferulate 5‐hydroxylase (HCT)
Glyma.04G040000	–	–	−2.08	N‐hydroxycinnamoyl transferase (HCT)
Glyma.01G004200	–	–	−1.32	Caffeoyl‐CoA O‐methyltransferase (CCoAOMT)
Glyma.05G147000	–	–	−1.54	Caffeoyl‐CoA O‐methyltransferase (CCoAOMT)
Glyma.19G007200	–	–	−1.12	Cinnamoyl‐CoA reductase (CCoAOMT)
Glyma.19G006900	–	–	−1.13	Cinnamoyl‐CoA reductase (CCoAOMT)
Glyma.09G201200	–	–	−2.11	Cinnamyl alcohol dehydrogenase (CAD)
Glyma.02G130400	–	–	−1.05	Chalcone synthase (CHS)
Glyma.09G075200	–	–	−1.13	Chalcone synthase (CHS)
Glyma.01G091400	–	–	−1.23	Chalcone synthase (CHS)
Glyma.08G110500	−1.26	−1.48	−2.05	Chalcone synthase (CHS)
Glyma.08G110700	−1.25	−1.27	−2.06	Chalcone synthase (CHS)
Glyma.08G109200	−1.3	−1.58	−2.13	Chalcone synthase (CHS)
Glyma.08G110400	−1.38	−1.64	−2.18	Chalcone synthase (CHS)
Glyma.08G110900	−1.12	−1.49	−2.18	Chalcone synthase (CHS)
Glyma.08G110300	−1.13	−1.5	−2.21	Chalcone synthase (CHS)
Glyma.16G219400	–	–	−2.23	NAD(P)H‐dependent 6′‐deoxychalcone synthase (DOCS)
Glyma.08G109300	−1.17	−1.53	−2.24	Chalcone synthase (CHS)
Glyma.08G109400	−1.3	−1.65	−2.42	Chalcone synthase (CHS)
Glyma.02G048700	–	–	1.49	Chalcone‐flavanone isomerase (CHI)
Glyma.07G150900	–	–	−2.50	Flavonol synthase (FLS)
Glyma.18G201900	–	–	−3.77	Flavonol synthase (FLS)
Glyma.18G267800	–	−5.27	−9.45	Flavonoid 4′‐O‐methyltransferase (FOMT)
Glyma.11G164700	–	–	2.99	Dihydroflavonol reductase (DHFR)
Glyma.12G238200	−0.88	−0.98	−2.07	Dihydroflavonol reductase (DHFR)
Glyma.11G027700	–	–	3.27	Anthocyanidin synthase (ANS)
Glyma.01G214200	–	–	2.95	Anthocyanidin synthase (ANS)
Glyma.02G226000	–	–	1.46	Anthocyanidin 3‐O‐glucosyltransferase (AGT)
Glyma.18G271600	3.31	–	–	Anthocyanin acyltransferase (AT)
Glyma.13G302300	–	–	2.49	Anthocyanin acyltransferase (AT)
Glyma.13G302500	−1.10		–	Anthocyanin 5‐aromatic acyltransferase (AT)
Glyma.14G034100	−5.85	−3.69	−2.93	Anthocyanin 5‐aromatic acyltransferase (AT)
Glyma.19G030800	–	–	4.58	Malonyl‐CoA:isoflavone 7‐O‐glucoside‐6′’‐O‐malonyltransferase (IFMaT)
Glyma.11G256500	–	–	4.61	Isoflavone 7‐O‐methyltransferase (IOMT)
Glyma.09G094400	–	−1.01	–	Isoflavone‐7‐O‐methyltransferase (IOMT)
Glyma.06G286600	–	–	2.69	Isoflavone 7‐O‐methyltransferase (IOMT)
Glyma.13G173300	–	–	−1.80	Isoflavone 4′‐O‐methyltransferase (IOMT)
Glyma.13G173600	–	–	−1.83	Isoflavone 4′‐O‐methyltransferase (IOMT)

a Fold changes (Log_2_FC) relative to S line.

In accordance with the transcriptomics data, we observed a marked accumulation of the metabolites phenylalanine, a phenylpropanoid pathway precursor and the lignin intermediates ferulic, benzoic, cinnamic and caffeic acids in the R line compared to the S line (Figure 4B). With the exception of caffeic acid, the accumulated metabolites markedly inhibited *S. sclerotiorum* growth *in vitro* (Figure S5). While caffeic acid did not significantly affect colony size, it clearly affected *S. sclerotiorum* growth patterns on PDA with the appearance of abnormal concentric ring growth patterns and premature sclerotia formation (Figure S5). Our data suggest a reprogramming of the phenylpropanoid pathway away from lignins and towards the accumulation of lignin intermediates, anthocyanins and phytoalexins in the resistance response against *S. sclerotiorum*. We propose that lignin intermediates, such as caffeic and ferulic acid, and possibly other compounds with antifungal activity are important components of this response.

### ROS scavenging and antioxidant activities are associated with resistance to *Sclerotinia sclereotiorum*

*Sclerotinia sclerotiorum*, via oxalic acid, is known to up‐regulate host ROS levels to induce PCD and achieve pathogenic success (Kabbage *et al*., 2013; Kim *et al*., 2008; Ranjan *et al*., 2018). Our transcriptomics analysis shows a differential regulation of genes related to ROS scavenging, such as peroxidases, glutathione S‐transferases (GSTs), ascorbate oxidases and superoxide dismutase (SODs), when comparing the R and S soybean lines (Table S8 and Figure S6). Three *GmGSTs* (Glyma.06G193400, Glyma.02G024600, Glyma.02G024800), two *GmSODs* (Glyma.12G081300, Glyma.12G178800) and five ascorbate oxidases or like proteins (Glyma.05G082700, Glyma.11G059200, Glyma.17G012300, Glyma.17G180400, Glyma.20G051900, Glyma.05G057400) were significantly up‐regulated in the R line compared to the S line as early as 24 hpi, suggesting a role in preventing oxidative damage imposed by *S. sclerotiorum* (Table S8 and Figure S6). Peroxidases were also differentially regulated at 24 hpi, with five family members (Glyma.01G19250, Glyma.11G049600, Glyma.11G080300, Glyma.14G201700, Glyma.17G177800) significantly up‐regulated; however, many others were down‐regulated in the R line compared to S line (Table S8 and Figure S6).

We mined our metabolomics data for differentially accumulated metabolites that may serve as ROS scavengers or antioxidants. Dehydroascorbic acid (DHA), the oxidized form of ascorbate, an important antioxidant, was specifically accumulated later in the infection time course (72 hpi), but not at the early stages in the R line (Figure S6). Similarly, the proline derivative, trans‐4‐hydroxy‐L‐proline, a known osmoprotectant and antioxidant (Hayat *et al*., 2012; Kim *et al*., 2017), is significantly accumulated in the R line at the later stages of the infection process (Figure S6). Proline plays a major role as an antioxidant, owing to its ROS scavenging capacity (Matysik *et al*., 2002; Smirnoff and Cumbes, 1989). Overall, these results and the earlier observation of anthocyanin induction point to a marked activation of ROS scavenging and antioxidant processes in the resistant response to *S. sclerotiorum*, presumably to counter to oxidative state imposed by this pathogen.

### Jasmonic acid signalling contributes to the resistant response to *Sclerotinia sclerotiorum*

**Figure 5:**
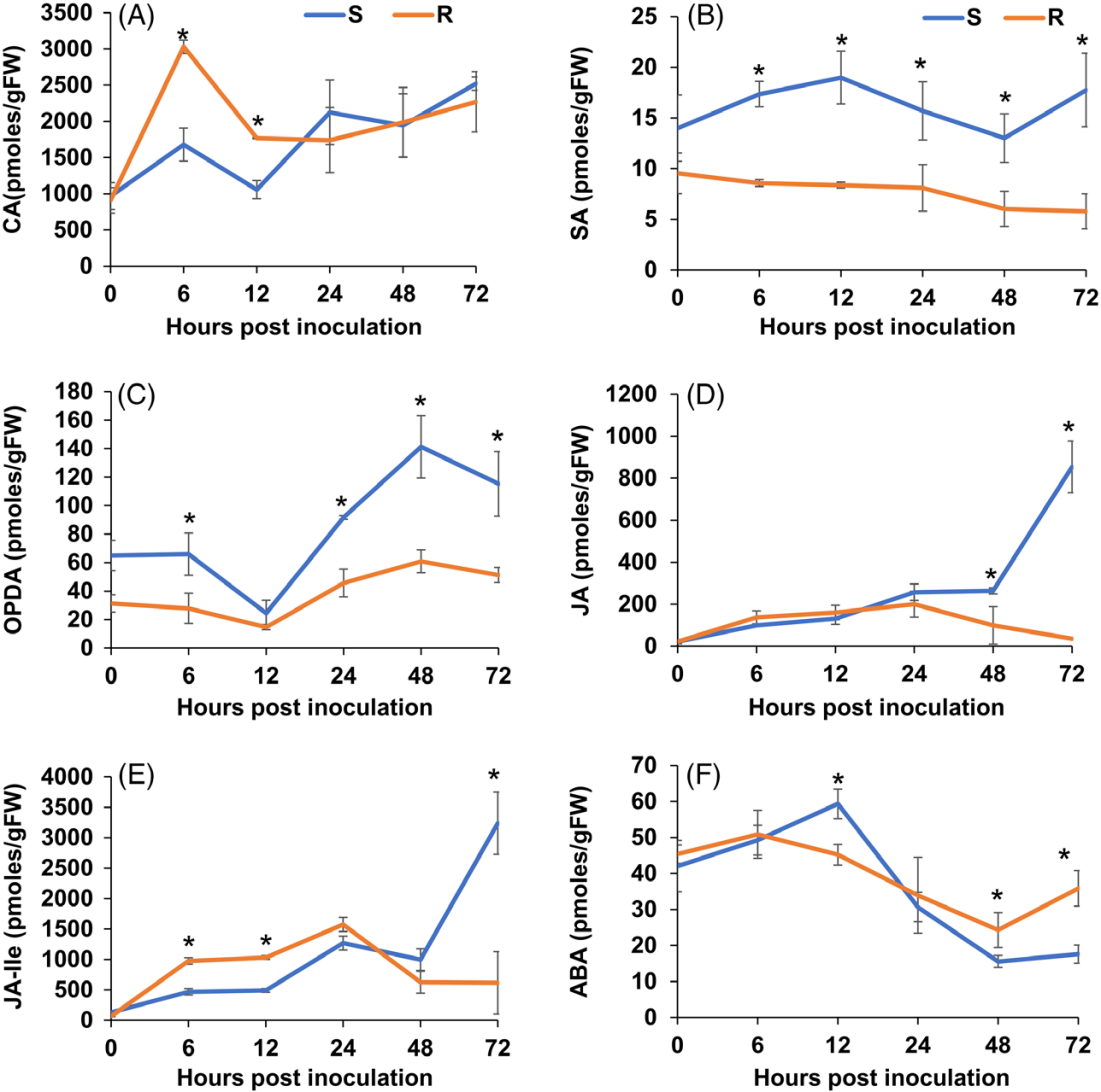
Estimation of phytohormones and a phenylpropanoid pathway precursor (cinnamic acid, CA) in R and S lines following *S. sclerotiorum* infection. (A) Cinnamic acid (CA), (B) salicylic acid (SA), (C) abscisic acid (ABA), (D) jasmonic acid (JA), (E) JA precursor 12‐oxophytodienoic acid (OPDA) and (F) bioactive JA derivative (+)‐7‐iso‐jasmonoyl‐L‐isoleucine (JA‐Ile). The bars represent the standard deviation (*n* = 3). *Indicates a significant difference between the R and S lines at *P*‐value <0.05 (one‐way ANOVA).

Our GO enrichment analysis highlighted DEGs between the R and S lines related to JA/ET biosynthesis and responses (Table S9 and Figure 2). Metabolic profiling also identified phytohormone‐related metabolites that were differentially accumulated during the course of infection between our lines, namely, linolenic acid, a precursor of jasmonic acid, and cyanoalanine, an indicator of ethylene biosynthesis (Figure S3). To confirm these results, we conducted a targeted GC‐MS analysis to more accurately estimate the dynamic changes in SA, ABA, cinnamic acid (an intermediate of SA and the phenylpropanoid pathway) and JA precursors and derivatives (12‐oxophytodienoic acid (OPDA) and (+)‐7‐iso‐jasmonoyl‐L‐isoleucine (JA‐Ile) in a time course experiment comparing the R and S soybean lines. Distinct patterns of phytohormone accumulation were identified during the course of infection (Figure 5). The bioactive form of JA, jasmonoyl‐L‐isoleucine (JA‐Ile) but not JA, was significantly induced as early as 6 hpi in the R line compared to the S line, before decreasing at the later stages of our time course (Figure 5E). Interestingly, JA and JA‐Ile levels drastically increased in the S line, albeit at a much later stage of infection (48–72 hpi). Thus, this late dramatic surge in JA and JA‐Ile in the S line may be perceived as a delayed response to fungal colonization, perhaps after the establishment of infection. In accordance, OPDA, a precursor of JA, accumulated at significantly higher levels in the S line at 48–72 hpi (Figure 5C). This pattern also explains the significant induction of JA biosynthetic transcripts at the later stages of infection observed in the S line (Table S9). Expectedly, SA accumulated to higher levels in the S line throughout the time course (Figure 5B). JA and SA responses are known to be antagonistic in many plant–pathogen interactions (Glazebrook, 2005). *In toto*, phytohormone estimation in the S and R lines following *S. sclerotiorum* challenge suggests that resistance to this pathogen in soybean coincides with an early induction of JA signalling. The timing of this induction appears to be critical to the outcome of this interaction.

### Antifungal activity and mode of action of stem extract from the resistant soybean line

Our earlier observation that soybean stems from the R line accumulated metabolites with antifungal activity, such as ferulic, benzoic, cinnamic and caffeic acids, following *S. sclerotiorum* challenge was intriguing. Thus, we reasoned that total stem extract from the resistant line should also exhibit antifungal activity against *S. sclerotiorum*. We prepared extracts from stem sections harvested from the R line following *S. sclerotiorum* inoculation that we termed red stem extracts due to the distinct red discoloration associated with resistant stems. *S. sclerotiorum* growth was assayed on potato dextrose broth (PDB) amended with the red stem extract, green stem extract (harvested from non‐inoculated plants) or DMSO control. Fungal biomass was markedly reduced (12‐ to 14‐fold) in the presence of the red stem extract compared to PDB amended with the green stem extract or DMSO (Figure S7). These results confirm that the resistant response associated with our R line clearly involves the accumulation of antifungal compounds that restrict *S. sclerotiorum* growth.

**Figure 6:**
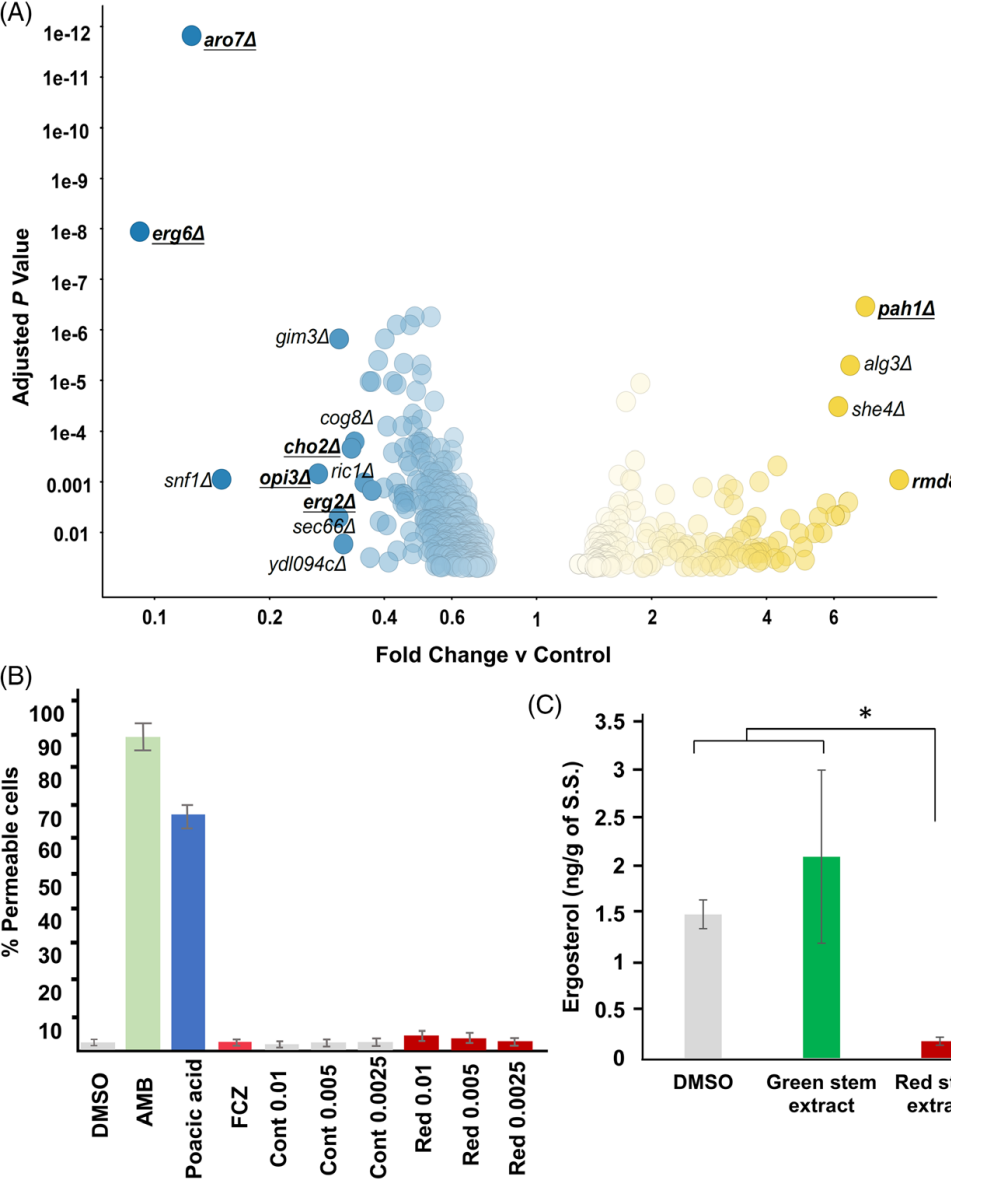
Chemical genomic profiling of the red stem extract in yeast mutants and ergosterol biosynthesis. (A) Plot showing growth differences between yeast deletion mutants exposed to the red stem extract. Dots represent mutants which were significantly sensitive (blue) or resistant (yellow) to the fungicidal activity of the extract. Underlined mutants are implicated in phospholipid and sterol biosynthesis (B) cell leakage assay to test the permeability of *S. sclerotiorum* treated with DMSO, fluconazole (FCZ, 1 mg/mL), amphotericin B (AMB, 100 μg/mL), poacic acid (500 μg/mL) and different dilutions of green (Cont) and red (Red) stem extract (% of concentrate), (C) ergosterol estimation from *S. sclerotiorum* treated with DMSO, green stem extract and red stem extract. Vertical bars show standard deviation of means of three replicates. *Indicates a significant difference at *P*‐value <0.05 (t‐test).

We next examined the mechanism by which the red stem extract inhibits fungal growth using chemical genomic profiling in yeast. Chemical genomic profiling is built upon barcoded strains of *Saccharomyces cerevisiae* mutants representing approximately 4000 gene deletions (Piotrowski *et al*., 2015). Deletion mutants of genes involved in phospholipid and sterol biosynthesis were significantly sensitive to the red stem extract (Fig. 6A). Mutants of *ERG6*, which encodes a protein in the ergosterol biosynthetic pathway, had the greatest sensitivity to the extract, and this was a highly significant response (*P* < 1e‐7). *ERG2*, which is also involved in ergosterol biosynthesis, was also significantly sensitive (*P* < 0.01). Mutants of *ARO7* which encode a gene involved in amino acid biosynthesis was significantly sensitive. While *ARO7* is not known to be directly involved in lipid/sterol biosynthesis, it has many genetic interactions with lipid‐related genes (Costanzo *et al*., 2016). *CHO2* and *OPI3* mutants were also significantly sensitive. Cho2p and Opi3p are both involved in phosphatidylcholine biosynthesis. A deletion mutant of *PAH1* was the most significantly resistant mutant. Pah1p is a phosphatase that regulates phospholipid synthesis. Deletion mutants of *PAH1* have increased phospholipid, fatty acid and ergosterol ester content (Pascual *et al*., 2013). Furthermore, in two of three replicates, the chemical genomic profile of the red stem extract had significant correlation (*P* < 0.05) with the profile of fenpropimorph (Parsons *et al*., 2006), an ergosterol biosynthesis inhibitor that targets *ERG2* and *ERG24* in yeast. Taken together, these data suggest that the red stem extract may exert toxicity by either disrupting enzymes involved in lipid/sterol biosynthesis or alternatively physically binding membrane lipids and causing cell leakage.

### Red stem extract inhibits ergosterol biosynthesis in *Sclerotinia sclerotiorum*

To test if the red stem extract causes rapid cell lysis like the antifungal compounds amphotericin B (binds ergosterol) and poacic acid (binds glucan), we performed a cell leakage assay. The red stem extract did not cause significant cell permeability and was similar to fluconazole, an ergosterol synthesis inhibitor (Figure 6B). This result supports the hypothesis that compounds within the red stem extract target ergosterol biosynthetic enzymes. We thus tested if the treatment of *S. sclerotiorum* with the red stem extract alters ergosterol production. *S. sclerotiorum* was grown in PDB amended with red stem extract, green stem extract or DMSO, and ergosterol levels were calculated as previously described (Yarden *et al*., 2014). Ergosterol content of *S. sclerotiorum* mycelia grown in red stem extract was significantly reduced compared to control treatments (Fig. 6C). The results confirm that the red stem extract inhibits the growth of *S. sclerotiorum* by targeting ergosterol biosynthesis in the fungus.

## Discussion

**Figure 7:**
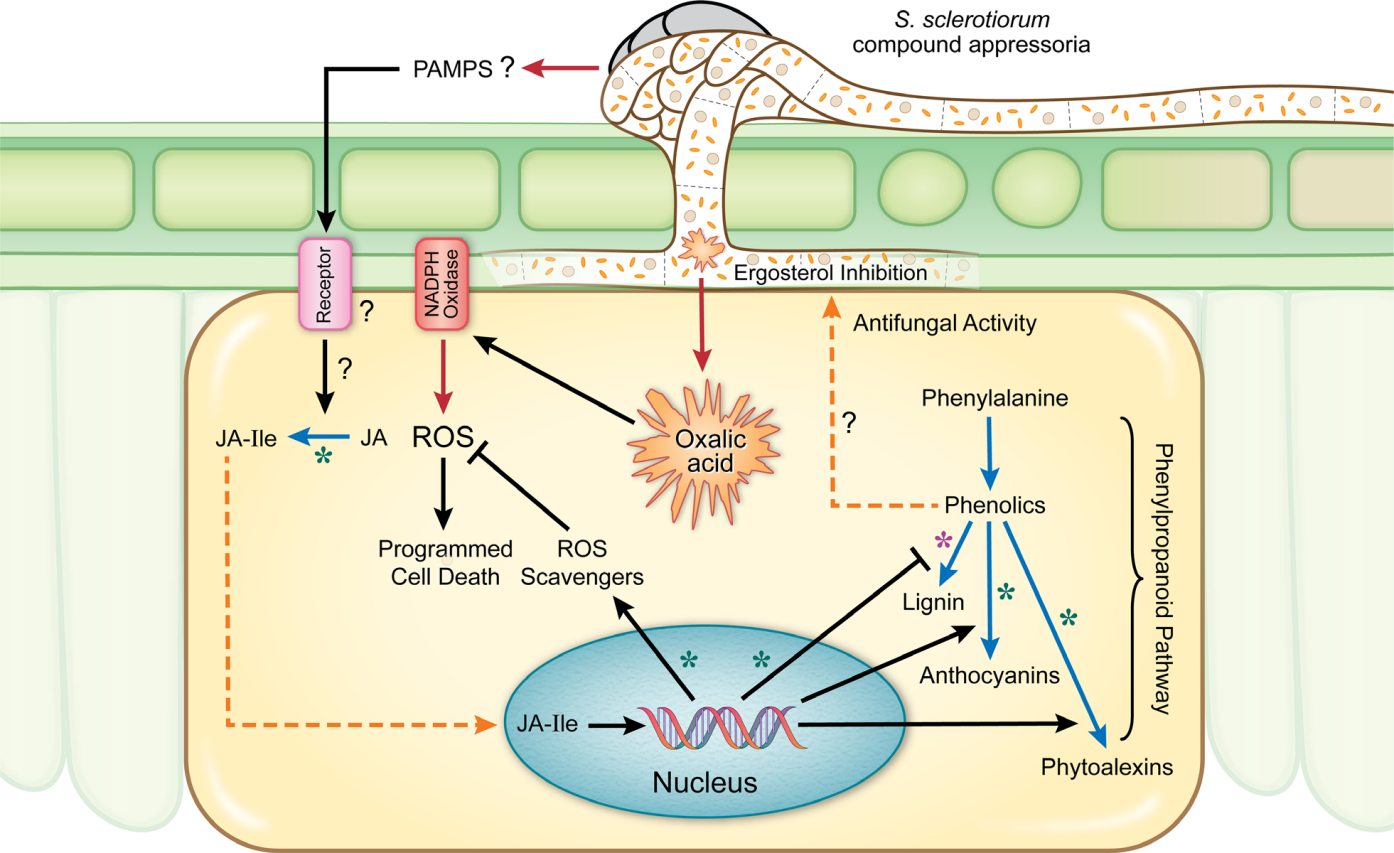
Cellular model summarizing *S. sclerotiorum* resistance mechanisms in soybean. Black line = induction/suppression of a process, red line = secretion/release, blue line = bioconversion, dashed orange line = translocation of a metabolite, purple asterisk (*) = down‐regulation in resistant response, green asterisk (*) = up‐regulation in resistant response, JA = jasmonic acid, JA‐Ile = jasmonic acid‐isoleucine.

Resistance to fungal pathogens with a predominately necrotrophic lifestyle, such as *Sclerotinia sclerotiorum*, is not well understood due to the likely complex network of responses to these pathogens or their determinants. Uncovering key components of these defence responses is essential for the deployment of disease‐resistant crops. In this study, we specifically examine soybean resistance mechanisms against *S. sclerotiorum* by comparing opposing outcomes in two soybean lines in response to this pathogen. Several lines of evidence are consistent with the following conclusions: (i) *S. sclerotiorum* challenge induces drastic changes in gene expression and metabolite production in soybean; (ii) resistance in soybean is associated with an early recognition of the pathogen and a rapid induction of JA signalling; (iii) the redox buffering capacity of the host is essential to counter the oxidative state imposed by *S. sclerotiorum*; (iv) a reprogramming of the phenylpropanoid pathway and up‐regulation of antifungal metabolites are observed during the resistant response to *S. sclerotiorum*; (v) the antifungal activity associated with resistance targets ergosterol biosynthesis in the pathogen. Overall, omics and functional studies allowed us to uncover a novel resistance mechanism connecting the up‐regulation of antifungal activity to a successful defence response against *S. sclerotiorum* and highlight the importance of early recognition and redox regulation in resistance to this pathogen (Figure 7).

The implication of host ROS in plant defences, including the hypersensitive response (HR) and pathogen‐associated molecular pattern (PAMP)‐triggered immunity following pathogen recognition, is well documented (Keinath *et al*., 2010; Zurbriggen *et al*., 2010). However, ROS are also produced during compatible interactions, thus facilitating host colonization of certain fungal pathogens (Gilbert and Wolpert, 2013; Kabbage *et al*., 2013; Ranjan *et al*., 2018; Williams *et al*., 2011). This study confirms the importance of ROS in the *S. sclerotiorum* pathosystem and suggests ROS scavenging and antioxidant activity as viable resistance mechanisms in soybean. We noted the significant up‐regulation of genes related to ROS scavenging such as peroxidases, glutathione S‐transferases, ascorbate oxidases and superoxide dismutase, in association with the successful defence response against *S. sclerotiorum*. We also noted the accumulation of antioxidant metabolites dehydroascorbic acid (DHA) and trans‐4‐hydroxy‐L‐proline. DHA is converted into ascorbic acid (AA), which is known for its redox buffering capacity and ROS detoxification (Foyer, 2005; Foyer and Noctor, 2005). The utilization of ascorbic acid as an antioxidant in cells causes its oxidation back to dehydroascorbic acid (Wilson and Wilson, 2002). Thus, the low levels of DHA in our resistant line at the onset of infection can be explained by higher ascorbic acid levels at this stage. Once oxidized, AA is converted to DHA, which accumulated at the later stages of our time course. Similarly, the proline derivative, trans‐4‐hydroxy‐L‐proline, was markedly increased in our resistance response and is a known osmoprotectant and antioxidant (Hayat *et al*., 2012; Kim *et al*., 2017). The accumulation of proline in plants has been implicated in stress tolerance by maintaining osmotic balance, stabilizing membranes and modulating ROS levels (Hayat *et al*., 2012). Overall, our accumulating evidence suggests that the antioxidant capacity of soybean plays a critical role in its ability to resist *S. sclerotiorum*, a pathogen that induces ROS and cell death in the host to achieve pathogenic success.

An important question is what causes this stark difference in ROS buffering capacity between the R and S soybean lines despite common genetic components? We propose that the early recognition of the pathogen in the R line leads to a timely response that includes the activation of the antioxidant machinery within the host. This is corroborated by our phytohormone analysis that shows a rapid induction of the bioactive form of jasmonic acid (JA), jasmonoyl‐L‐isoleucine (JA‐Ile) as early as 6 hpi in the resistance response, JA‐Ile levels decreased at the later stages of infection, presumably once the infection was under control. In contrast, in the susceptible response, JA and JA‐Ile levels remained low early, but drastically increased at the later stages of our time course, which can be conceived as a delayed and unsuccessful response to fend off an already established infection. Plant defences involving JA typically inhibit fungal necrotrophs (Glazebrook, 2005), and mutants specifically impaired in JA‐Ile accumulation show enhanced susceptibility to such pathogens (Agarwal *et al*., 2011; Gravot *et al*., 2012; Yan and Xie, 2015). JA and ethylene pathways were also suggested to be involved in soybean–*S. sclerotiorum* interaction (Calla *et al*., 2009). Our results are consistent with the model where upon *S. sclerotiorum* challenge, JA is rapidly biosynthesized from linolenic acid and subsequently catalysed to JA‐Ile, the active form of JA. Our metabolomics analysis also showed that linolenic acid is hyperaccumulated early in the resistance response. Thus, while JA signalling appears to be activated in both resistant and susceptible responses, the timing of this induction is key to the resistance outcome in *S. sclerotiorum*–soybean interaction. The early induction likely leads the timely activation of defence components culminating in the arrest of fungal growth and colonization.

The integration of our transcriptomic and metabolic data revealed that the phenylpropanoid pathway is differentially regulated in our soybean lines in response to *S. sclerotiorum* challenge. Specifically, our data suggest a reprogramming of the phenylpropanoid pathway with its flux diverted from lignin to lignin intermediates, anthocyanins and phytoalexins in the resistance response against *S. sclerotiorum*. While these results will require further confirmation by the absolute quantification of phenylpropanoid pathway components, the accumulation of upstream metabolites such as ferulic acid, caffeic acid, cinnamic acid and benzoic acid in the resistance line supports a reduced flow towards lignins. The observed accumulation of ferulic acid is also consistent with decreased lignification considering its role as a nucleation site for lignin polymerization (Grabber *et al*., 2002). These results may seem counterintuitive considering that lignin biosynthesis is associated with cell wall fortification as a mechanism of disease resistance (Vance, 1980). However, in support of our results, a negative correlation between soybean stem lignin content and resistance to *S. sclerotiorum* has previously been reported (Peltier *et al*., 2009). Flux changes within the phenylpropanoid pathway in maize have been discussed in response to the biotrophic fungal pathogen *Ustilago maydis* (Djamei *et al*., 2011; Tanaka *et al*., 2014). In contrast to our results, resistance to *U. maydis* appears to be associated with the activation of the lignin branch of the phenylpropanoid pathway. However, these observations are in line with the limited lytic repertoire and the biotrophic lifestyle of *U. maydis*. Against necrotrophic fungal pathogens, anthocyanin may act as antioxidants by scavenging ROS and limiting the induction of cell death required by these pathogens. Indeed, the ROS scavenging capacity of anthocyanins has been proposed to provide protection against necrotrophic pathogens, including *S. sclerotiorum* (Calla *et al*., 2009), *Botrytis cinerea* (Zhang *et al*., 2013) and *Erwinia carotovora* (Lorenc‐Kukuła *et al*., 2005). Our results confirm that this mechanism may confer resistance against *S. sclerotiorum* in soybean.

Due to the observed accumulation of phenylpropanoid metabolites in the resistance response against *S. sclerotiorum*, we considered antifungal activity as a component of this response. Indeed, total red stem extracts from our resistant line following *S. sclerotiorum* challenge clearly inhibited fungal growth *in vitro*. This antifungal response was seemingly absent from healthy soybean plants, activated only in response to *S. sclerotiorum*. Furthermore, we were able to show using chemical genomics in yeast that this antifungal activity targets ergosterol biosynthesis in the fungus. Ergosterol, a lipid found in the cellular membranes of fungi, is important to the regulation of membrane fluidity and permeability. It is, therefore, conceivable that plants have evolved means to target this key component of fungal membranes. The specific metabolites responsible for these antifungal activities in our resistant line are currently unknown. While we provided evidence that several identified metabolites affected *S. sclerotiorum* growth, their chemical genomic profile did not match that of the red stem extract. Thus, we propose that other unknown compounds target ergosterol biosynthesis and contribute to resistance to this pathogen. The identification of these compounds through high‐resolution mass spectrometry may lead to the discovery of novel bioactive metabolites and help devise specific strategies to introgress resistance to fungal pathogens in crop plants.

## Methods

### Plant material and pathogen inoculation

Two recombinants inbred lines of soybean (*Glycine max*), one resistant (91‐145) and one susceptible (91‐144), were used in this study. SSR infection was performed using a wild‐type strain of *S. sclerotiorum* (1980) grown at room temperature on potato dextrose agar (PDA) as described by Godoy *et al*., 1990. Plants were *grown* in a growth room at 24°C with 16 h light and 8 h dark photoperiod cycles. Fertilization was applied using standard practices. Four‐week‐old soybean plants were infected with *S. sclerotiorum* by petiole inoculation, using an agar plug of actively growing fungal hyphae. Plant tissue was sampled by cutting horizontally above and below (1.5 cm) the node of the inoculated petiole with a clean straight‐edge razor (Ranjan *et al*., 2018). Tissue samples were then immediately frozen in liquid nitrogen prior to RNA extraction and metabolomic analysis. Samples from non‐inoculated stem tissues were harvested as above and served as a control. The experimental design was completely randomized and consisted of three biological replicates for each of the treatments. Each biological replicate consisted of stem segments (∼3 cm, first internode) from two different plants.

### RNA extraction and library preparation

Total RNA was extracted from soybean stem tissues using a modified Trizol protocol (Invitrogen Corp., Carlsbad, CA, USA). Briefly, collected tissue from each sample was finely ground in liquid nitrogen. For each 100 mg of tissue, 1 ml of chilled Trizol was added. Samples were centrifuged at 12 k rpm for 5 min at 4°C. Supernatant was discarded and 200 μl of chilled chloroform was added and vortexed at high speed for 15 s. Samples were centrifuged again at 12 k rpm for 15 min at 4°C. The aqueous phase was mixed with 0.8× isopropanol and left at room temperature for 10 min, followed by centrifugation at 12 k rpm at 4°C. Supernatant was discarded, and the pellet was washed with 75% ethanol. Pellet was air‐dried for 10 min at room temperature and resuspended in 20 μL of nuclease‐free water followed by incubation at 55°C for 10 min. Samples were cleaned using the RNeasy Plant Mini Kit (Qiagen, Hilden, Germany). RNA concentration and purity were determined by Nanodrop (Thermo Fisher Scientific, Wilmington, DE) and sample quality was assessed using an Agilent Bioanalyzer 2100 and an RNA 6000 Nano Kit (Agilent Technologies, Santa Clara, CA). The RNAseq experiment included three biological replicates per treatment.

Library preparation was performed at the University of Wisconsin–Madison Biotechnology Centre (Madison, WI, USA). Individually indexed libraries were prepared using the TruSeq RNA Sample Preparation v2 kit according to the manufacturer's instructions (Illumina, San Diego CA, USA). Library concentrations were quantified with the Qubit HS DNA kit (Thermo Fisher Scientific, Wilmington, DE). The size and quality of the libraries were evaluated with an Agilent Bioanalyzer 2100 and an Agilent DNA 1000 kit (Agilent Technologies, Santa Clara, CA) and the libraries were sequenced using Illumina HiSeq2500 (1 × 100 bp) (Illumina, San Diego CA, USA).

### Quality check and sequence analysis

Illumina raw read data quality was verified with fast QC (Andrews, 2010) and quality control checked using FASTX‐toolkit (Blankenberg *et al*., 2010). The soybean and *S. sclereotiorum* genome sequences were acquired from Phytozome v12.1 (https://phytozome.jgi.doe.gov/pz/portal.html#!bulk?org=Org_Gmax) and the Broad institute (https://www.broadinstitute.org/fungal-genome-initiative/sclerotinia-sclerotiorum-genome-project) respectively (Amselem *et al*., 2011; Schmutz *et al*., 2010). Raw sequence reads were mapped to both genomes using the Subjunc aligner from Subread (Liao *et al*., 2013). Alignments were compared to the gene annotation GFF files for both organisms (Soybean: Gmax_275_Wm82.a2.v1.gene.gff3 (Schmutz *et al*., 2010), *S. sclereotiorum*: sclerotinia_sclerotiorum_2_transcripts.gtf (Amselem *et al*., 2011)) and raw counts for each gene were generated using the feature Counts tool from subread. The raw counts data were normalized using voom from the R Limma package, then used to generate differential expression (log_2_FC) values (Law *et al*., 2014; Ritchie *et al*., 2015). DEGs were generated from the comparison of inoculated soybeans of both lines at different time points to their respective uninoculated control (FDR < 0.05; log_2_FC > 1 or <−1). Raw data were submitted to NCBI (Accession: GSE114448).

### Gene annotation and gene ontology enrichment analysis

Differentially expressed genes were annotated using soybean genome gene annotations (Annotation_Gmax_275_Wm82.a2.v1.gene.gff3) from Phytozome (https://phytozome.jgi.doe. gov/pz/portal.html#!bulk?org=Org_Gmax) while soybean chloroplast (https://www.ncbi.nlm.nih.gov/nuccore/91214122) and mitochondrion (https://www.ncbi.nlm.nih.gov/nuccore/476507670) sequences were used from NCBI. The statistically significant DEGs (*P*‐value < 0.01; log_2_FC > 1 or <−1) of the R versus S comparison were used to identify enriched Gene ontology (GO) terms using the Soybase GO term enrichment tool (https://www.soybase.org/goslimgraphic_v2/dashboard.php) (Morales *et al*., 2013).

### Metabolites estimation and analysis

Four‐week‐old soybean plants of susceptible and resistant lines were petiole inoculated as described above. Four stems (3 cm) for each treatment per biological replicate were harvested 48 and 72 h post‐inoculation and non‐inoculated stems were used as a control. Collected stem samples were immediately frozen in liquid nitrogen and kept at −80°C until used. Gas chromatography–mass spectrometry (GC–MS) analysis was performed by West Coast Metabolomics (Univ. California, Davis). Detailed methods of metabolite derivatization, separation and detection are described in Fiehn, 2016; . Briefly, samples were injected (0.5 μL, split less injection) into a Pegasus IV GC (Leco Corp., St Joseph, MI) equipped with a 30 m × 0.25 mm i.d. fused‐silica capillary column bound with 0.25 μm Rtx‐5Sil MS stationary phase (Restek Corporation, Bellefonte, PA). The injector temperature was 50°C ramped to 250°C by 12°C/s. A helium mobile phase was applied at a flow rate of 1 mL/min. Column temperature was 50°C for 1 min, ramped to 330°C by 20°C/min and held constant for 5 min. The column effluent was introduced into the ion source of a Pegasus IV TOF MS (Leco Corp., St Joseph, MI) with transfer line temperature set to 230°C and ion source set to 250°C. Ions were generated with a −70 eV and 1800 V ionization energy. Masses (80–500 m/z) were acquired at a rate of 17 spectra s‐1. ChromaTOF 2.32 software (Leco Corp) was used for automatic peak detection and deconvolution using a 3 s peak width. Peaks with signal/noise below 5:1 were rejected. Metabolites were quantified by peak height for the quantification ion. Metabolites were annotated with the BinBase 2.0 algorithm (Skogerson *et al*., 2011). Statistical analyses were conducted in MetaboAnalyst 3.0 (Xia *et al*., 2015). Multivariate and univariate statistics were performed on generalized log transformed peak heights. The multivariate analysis of identified metabolites was performed using Partial Least Squares‐Discriminant Analysis (PLS‐DA). Metabolites with FDR < 0.05 were considered differentially regulated. Metabolite pathway analysis was done using the MetaboAnalyst 3.0 Pathway Analysis tool (Xia *et al*., 2015).

### Reverse Transcriptase–quantitative PCR

RNA was isolated as described above. RNA was reverse transcribed using the AMV First‐Strand cDNA Synthesis Kit (NEB Inc., Ipswitch, MA) according to manufacturer's instructions. RT‐qPCR was performed using a SensiFAST SYBR No ROX Kit (Bioline USA Inc., Taunton, MA, USA). Primers were designed using Primer3 software (Koressaar and Remm, 2007; Untergasser *et al*., 2012) for the amplification of gene fragments of approximately 100–200 bp in length and with an annealing temperature of 60°C (Table S7). RT‐qPCR was performed on a CFX96 real‐time PCR system (Bio‐Rad, Hercules, CA). The run conditions were 2 min of initial denaturation at 95°C, 95°C for 5 s, 58°C for 10 s and 72°C for 20 s (40 cycles). The relative expression of genes was calculated using the 2^−ΔΔCt^ method (Livak and Schmittgen, 2001) with the soybean gene *GmCon15S* (Libault *et al*., 2008) as endogenous control. Three biological replicates were used for each sample.

### Targeted GC‐MS analysis of CA, SA, JA, JA‐Ile, cis‐OPDA and ABA

Four‐week‐old soybean plants of susceptible and resistant lines were petiole inoculated with actively growing agar plugs of *S. Sclerotiorum*. Four stems (3 cm) for each biological replicate were harvested at 6, 12, 24, 48 and 72 hpi. Uninoculated stems were also collected for the estimation of the basal concentration of the phytohormones. These collected stem samples were immediately frozen with liquid nitrogen and kept at −80°C until used. Stem tissues from each treatment were finely ground with liquid nitrogen and 100 mg of the ground tissue was added to 500 μl of phytohormone extraction buffer and analysed using GC‐MS (Christensen *et al*., 2014; Schmelz *et al*., 2003, 2011). The simultaneous detection of several hormones was accomplished using the methods described in Muller *et al*. 2011 with modifications (Müller and Munné‐Bosch, 2011). The analysis utilized an Ascentis Express C‐18 Column (3 cm × 2.1 mm, 2.7 μm) connected to an API 3200 LC‐electrospray ionization‐tandem mass spectrometry (MS/MS) with multiple reaction mentoring (MRM). Three biological replicates of each treatment were performed.

### Plate inhibition assay of *S. sclerotiorum*

The plate growth inhibition assay of *S. sclerotiorum* was done on solid PDA culture plates containing 0, 250, 500 or 1000 μg/mL of ferulic or caffeic acid. Three replicates were used for each treatment. Plates were inoculated with an actively growing plug of *S. sclerotiorum* and grown at 25 °C for either 48 h (ferulic acid) or 7 days (caffeic acid) prior to assessment.

### Compound extraction from soybean stem

Five hundred milligram of infected red stem or unaffected green stem was mixed 1:1 w/v in 100% ethanol at 80°C for 1 h. Samples were resuspended with 100 μL of DMSO and used for *S. sclerotiorum* inhibition assay, chemical genomics and cell permeability assays.

### Chemical genomic analysis

Chemical genomic analysis of the red stem extract was performed using the non‐essential yeast deletion mutant collection as described previously (Piotrowski *et al*., 2015). Briefly, triplicate 200 μL cultures of the pooled deletion collection were exposed to a 1:10 dilution of the red stem extract and allowed to grow for 48 h. Genomic DNA was extracted using the Invitrogen Purelink 96‐well genomic extraction kit (Invitrogen, Carlsbad, CA, USA). Mutant‐specific barcodes were amplified using indexed primers. Samples were sequenced on a HiSeq2500 (1 × 50 bp) (Illumina, San Diego CA, USA) rapid run and reads were processed using BEAN‐counter (Piotrowski *et al*., 2017) and EdgeR (Robinson *et al*., 2009).

### Cell permeability assay

To quantify the membrane damage caused by the red stem extract, a FungaLight™ Cell Viability assay (Invitrogen L34952) and Guava Flow Cytometer (Millipore, USA) were used as described previously (Wyche *et al*., 2014). Amphotericin B (100 μg/mL) and poacic acid (500 μg/mL) were included as positive controls. Fluconazole (1 mg/mL) was also included as a control, given its ability to inhibit ergosterol biosynthesis without causing rapid cell permeability. We exposed 200 μL log phase populations of yeast cells in YPD media to the control drugs, red stem extract (0.01%, 0.005% and 0.0025%), the control green stem extract (0.01%, 0.005% and 0.0025%) and a 1% DMSO control (*n* = 3) for 4 h at 30°C. The cells were then stained with the FungaLight™ kit and immediately analysed by flow cytometry.

### Biomass and ergosterol estimation of *S. sclerotiorum*

The biomass of *S. sclerotiorum* was measured by growing the fungus in potato dextrose broth (PDB). Freshly grown PDA cultures were scraped and then washed twice with water at 4000 rpm (4°C) before being resuspended in water. Equal amounts of resuspended *S. sclerotiorum* were inoculated into 250 mL conical flasks containing 30 mL PDB. For each 30 mL of PDB, 300 μL of red stem extract, green stem extract or DMSO was added and incubated over a period of 48 h. To estimate the wet weight, the mycelia were filtered on a preweighed Miracloth (Darmstadt, Germany). Ergosterol level was estimated as described by Yarden et. al 2014 (Yarden *et al*., 2014).

## Conflict of interest

The authors declare no conflicts of interest.

## Supporting information


**Figure S1** Differentially expressed genes (DEGs) identification in R and S line after *S. sclerotiorum* infection.
**Figure S2** Partial least squares‐discriminate analysis (PLS‐DA) score plots of metabolic profiles in soybean R and S line.
**Figure S3** (A) Increased accumulation of the jasmonic acid precursor linolenic acid in the R line compared to the S line; (B) increased accumulation of cyanoalanine (an indicator of ethylene biosynthesis) in the R line compared to the S line.
**Figure S4** Confirmation of expression profiles of select phenylpropanoid pathway genes using qRT‐PCR.
**Figure S5** Effect of phenylpropanoid pathway intermediate on *S. sclerotiorum* growth.
**Figure S6** (A) Red and green stem extract of a R line plant infected with *S. sclerotiorum* and a S line plant mock inoculated. Extraction was performed 10 dpi; (B) fungal biomass after growth in PDB cultures containing the red stem extract, DMSO or green stem extract; (C) weight of fungal biomass in PDB cultures containing the red stem extract, green stem extract or DMSO.
**Figure S7** Reactive oxygen species (ROS) scavenging machinery.

**Table S1** Differentially expressed genes (DEGs) in R and S lines following *S. sclerotiorum* infection at 24, 48 and 96 hpi compared to control.

**Table S2** Differentially expressed genes in the R line compared to the S line following *S. sclerotiorum* infection at 24, 48 and 96 hpi.

**Table S3** GO enrichment of significant biological processes generated from differentially regulated genes in the R line compared to the S line.

**Table S4** Estimated gas chromatography–mass spectrometry (GC–MS) peak intensity list of all the metabolites.

**Table S5** Significantly regulated metabolites in the R line compared to the S line following *S. sclerotiorum* infection at 24, 48 and 96 hpi.

**Table S6** Metabolic pathways assigned to significantly regulated metabolites from comparison of R and S lines at 48 and 72 hpi.

**Table S7** Primer list for qRT‐PCR of phenylpropanoid genes.

**Table S8** Differentially expressed genes encoding putative reactive oxygen species (ROS) scavenging and antioxidant genes in the R line compared to the S line following *S. sclerotiorum* infection at 24, 48 and 96 hpi.

**Table S9** Differentially expressed genes encoding putative jasmonic acid (JA) and ethylene (ET) biosynthetic and response genes in the R line compared to the S line following *S. sclerotiorum* infection at 24, 48 and 96 hpi.
