# Supplementary material for: Resistance against Sclerotinia sclerotiorum in soybean involves a reprogramming of the phenylpropanoid pathway and up‐regulation of antifungal activity targeting ergosterol biosynthesis
Source: Plant Biotechnol J. 2019 Feb 11;17(8):1567–81. doi: 10.1111/pbi.13082 (PMC6662107; doi:10.1111/pbi.13082)
Supplement: Supplementary file 1 — Figure S1 Differentially expressed genes (DEGs) identification in R and S line after S. sclerotiorum infection. Figure S2 Partial least squares‐discriminate analysis (PLS‐DA) score plots of metabolic profiles in soybean R and S line. Figure S3 (A) Increased accumulation of the jasmonic acid precursor linolenic acid in the R line compared to the S line; (B) increased accumulation of cyanoalanine (an indicator of ethylene biosynthesis) in the R line compared to the S line. Figure S4 Confirmation of expression profiles of select phenylpropanoid pathway genes using qRT‐PCR. Figure S5 Effect of phenylpropanoid pathway intermediate on S. sclerotiorum growth. Figure S6 (A) Red and green stem extract of a R line plant infected with S. sclerotiorum and a S line plant mock inoculated. Extraction was performed 10 dpi; (B) fungal biomass after growth in PDB cultures containing the red stem extract, DMSO or green stem extract; (C) weight of fungal biomass in PDB cultures containing the red stem extract, green stem extract or DMSO. Figure S7 Reactive oxygen species (ROS) scavenging machinery. [file PBI-17-1567-s010.pdf]

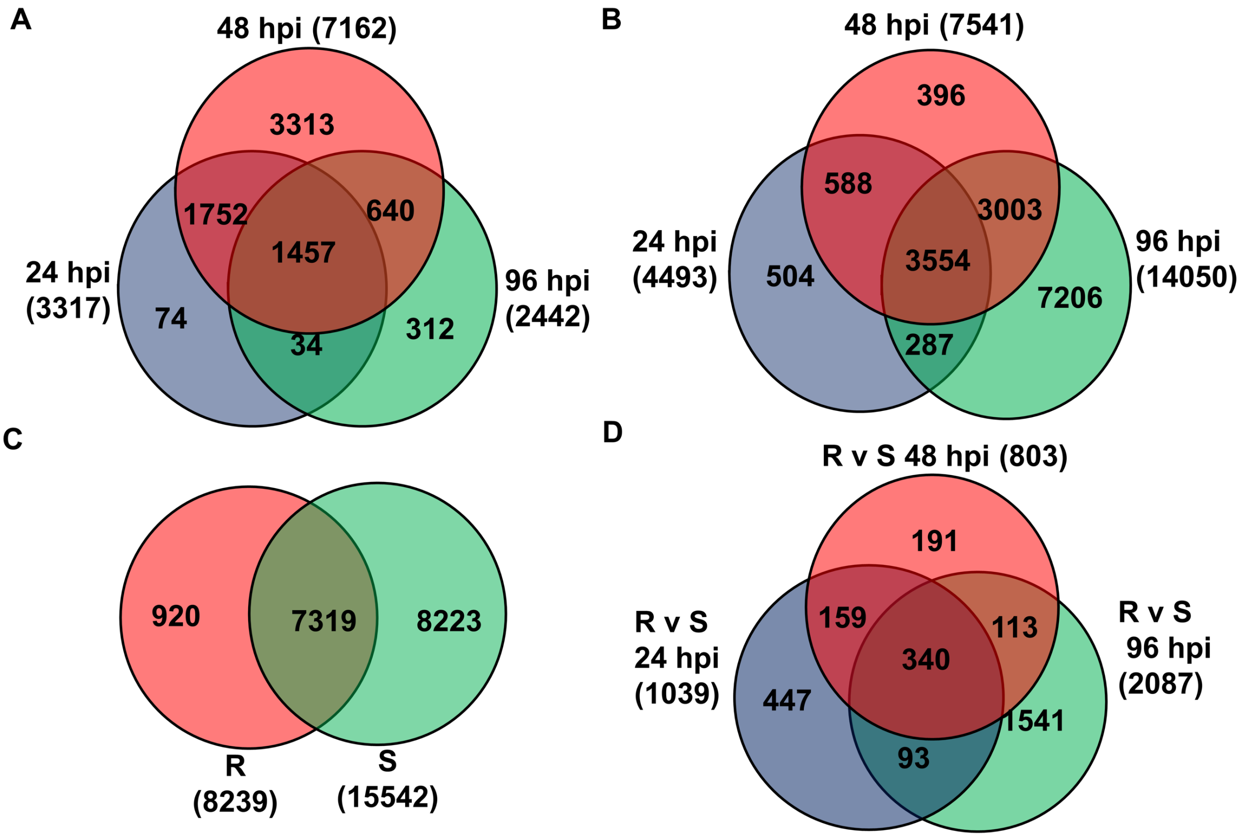

Supplementary Figure 1. Differentially expressed genes (DEGs) identification in R and S line after *S. sclerotiorum* infection. Venn diagram showing (A) DEGs in R line at 24, 48 and 96 hpi compared to control (non-inoculated) sample, (B) DEGs in S line at 24, 48 and 96 hpi compared to control (non-inoculated) sample, (C) In total, 921 and 8223 DEGs were unique to the R and S line, respectively, while 7319 were identified in both the lines, (D) DEGs in R line compared to S line at 24, 48 and 96 hpi.

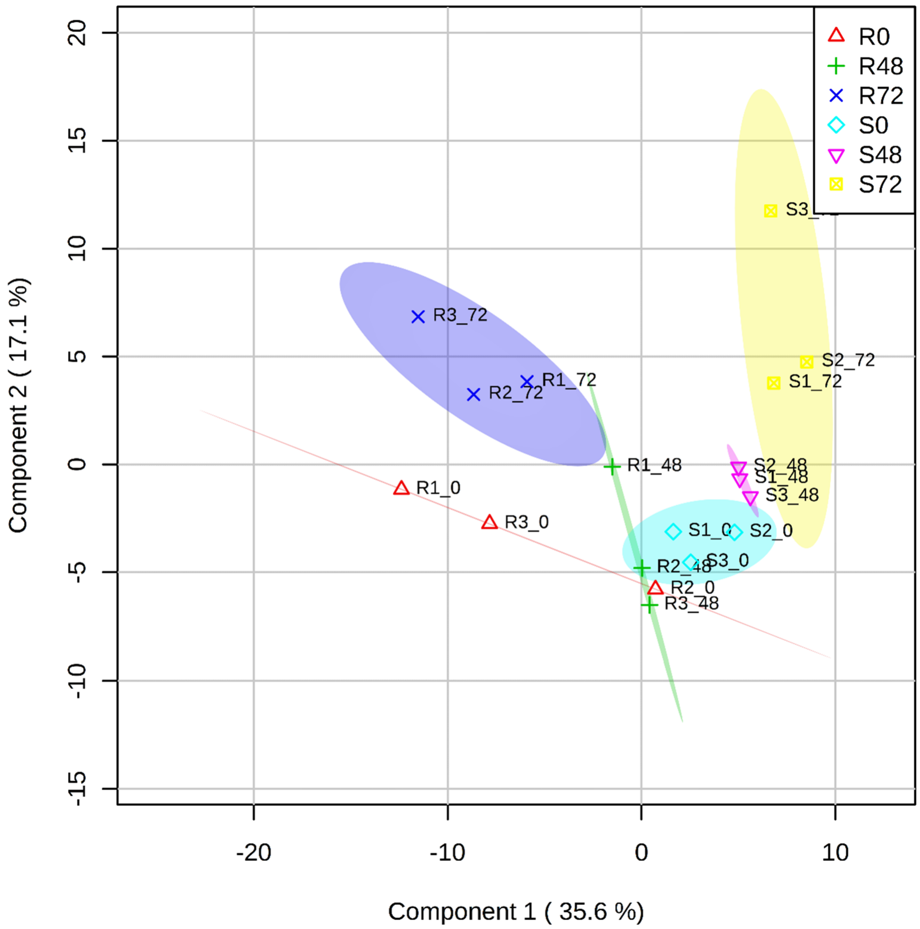

Supplementary Figure 2. Partial least squares-discriminate analysis (PLS-DA) score plots of metabolic profiles in soybean R and S line. The first (PC1) and second (PC2) principal components explain 52.7% of the variance. Control samples are R0 and S0. *S. sclerotiorum* infected samples at 24, 48 and 96 hpi for the R lines are represented as R24, R48, and R96, respectively. *S. sclerotiorum* infected samples at 24, 48 and 96 hpi for the S line are represented as S24, S48, and S96, respectively. Numbers 1, 2, and 3 represents three independent biological replicates.

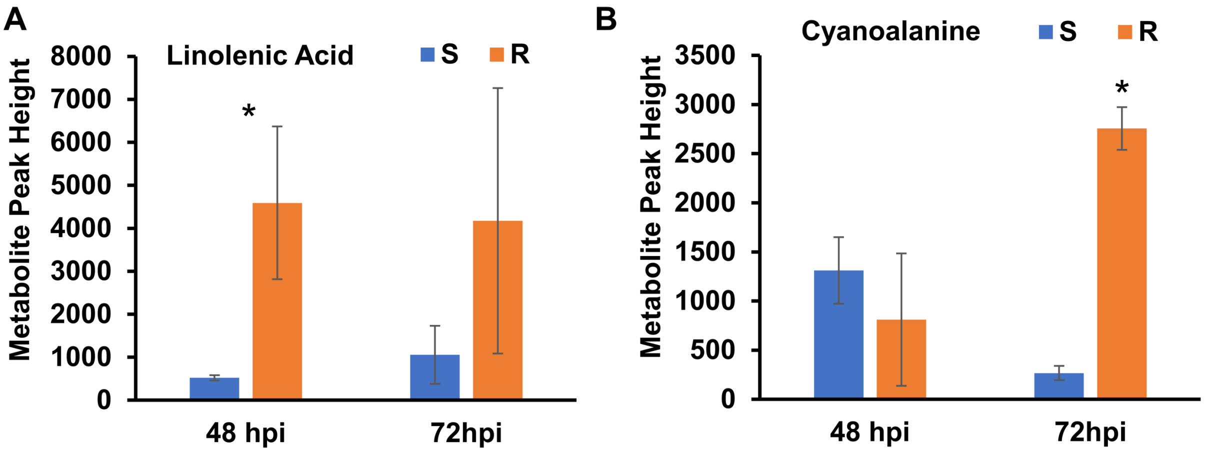

Supplementary Figure 3. (A) Increased accumulation of the jasmonic acid precursor linolenic acid in the R line compared to the S line, (B) Increased accumulation of cyanoalanine (an indicator of ethylene biosynthesis) in the R line compared to the S line. The bars represent the standard deviation (n = 3). * Indicates a significantly difference at p-value < 0.05 (t-test).

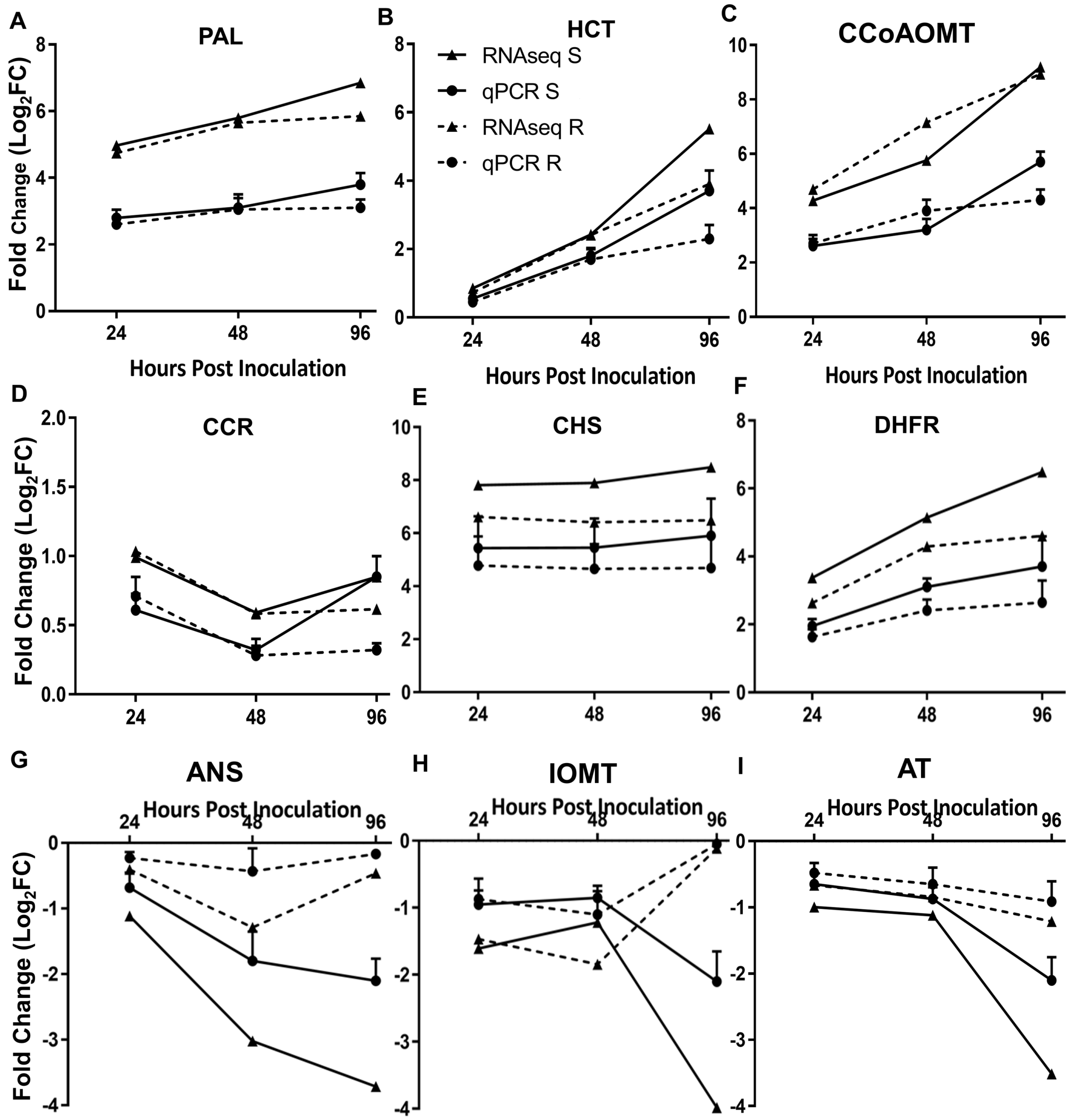

Supplementary Figure 4. Confirmation of expression profiles of select phenylpropanoid pathway genes using qRT-PCR. (A) PAL (Phenylalanine ammonia-lyase, Glyma.02G309300); (B) HCT (N-hydroxycinnamoyl transferase, Glyma.04G040000); (C) CCoAOMT (Caffeoyl-CoA O-methyltransferase, Glyma.05G147000); (D) CCR (Cinnamoyl-CoA reductase, Glyma.19G006900); (E) CHS (Chalcone synthase, Glyma.08G110400); (F) DHFR (Dihydroflavonol reductase, Glyma.12G238200); (G) ANS (Anthocyanidin synthase, Glyma.11G027700); (H) IOMT (Isoflavone 7-O-methyltransferase, Glyma.11G256500) and (I) AT (Anthocyanin acyltransferase, Glyma.18G271600). The fold changes in expression values for qRT-PCR were calculated by comparing the expression values of genes in inoculated vs. non-infected soybean stem tissues using the $2^{-\Delta\Delta Ct}$ method. GmCons15 was used as endogenous control. The absolute fold changes were converted to Log_2FC. Data are presented as means ± standard deviation (SD) from three independent experiments.

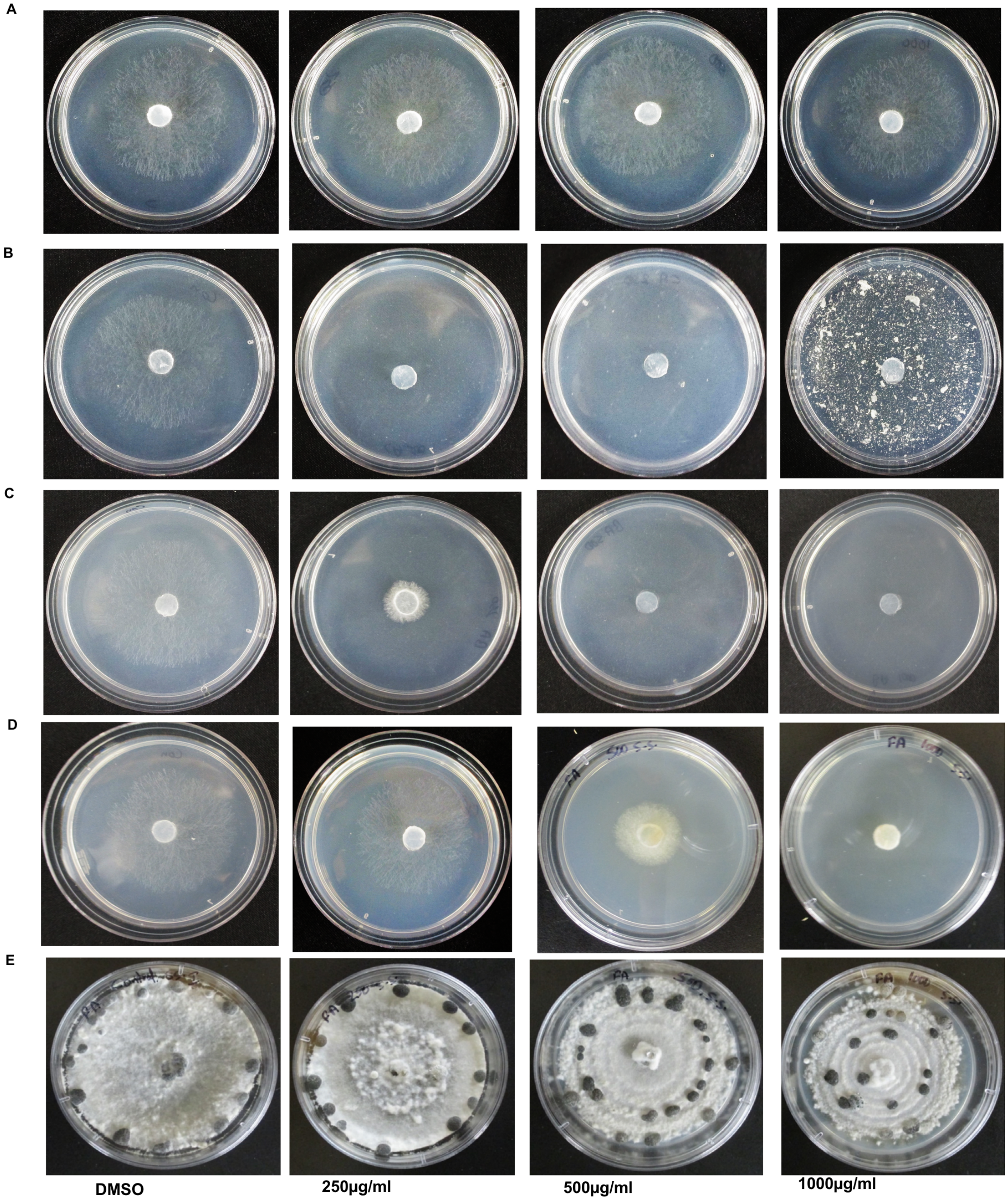

Supplementary Figure 5. Effect of phenylpropanoid pathway intermediate on *S. sclerotiorum* growth. Cinnamic acid (B), Benzoic acid (C), Ferulic acid (D) inhibits the growth of *S. sclerotiorum* while Phenylalanine (A), does not. Representative photographs (A,B,C and D) were taken 24 hours post inoculation. Caffeic acid (E) affects normal development of *S. sclerotiorum*. Representative photographs were taken 7 days post inoculation. DMSO (Dimethyl sulfoxide) is the solvent control. Concentrations of the compounds are in µg/ml

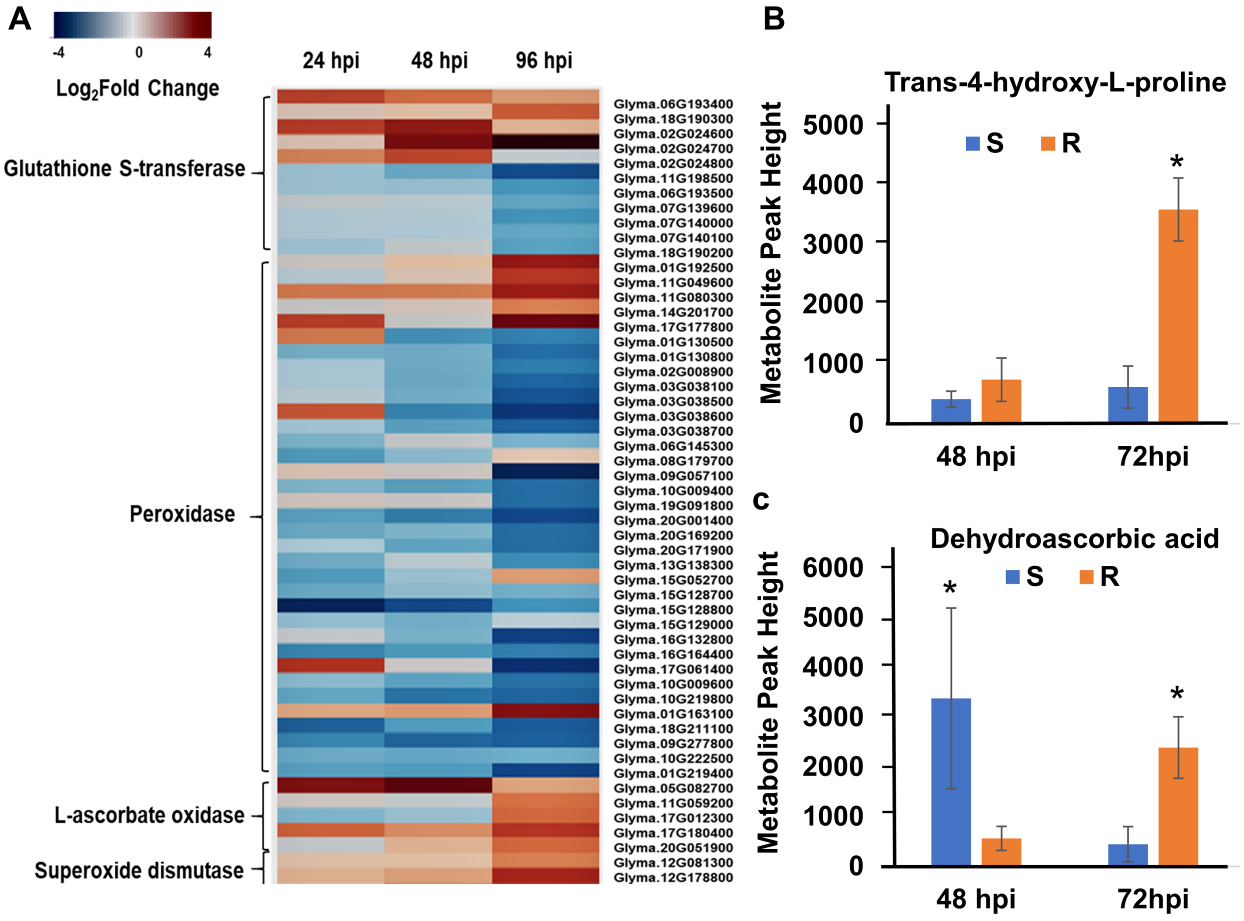

Supplementary Figure 6. Reactive oxygen species (ROS) scavenging machinery. (A) Heat map of ROS scavenging (Glutathione S-transferase, Peroxidase, L-ascorbate oxidase, and Superoxide dismutase) and antioxidant genes (Proline-rich protein) induced in the R line compared to the S line during *S. sclerotiorum* infection at 24, 48 and 96 hpi. (B) Differential accumulation of ROS related metabolites trans-4-hydroxy-L-proline and dehydroascorbic acid during *S. sclerotiorum* infection at 48 and 72 hpi. Data are presented as means ± standard deviation (SD) from three independent experiment. * Indicates a significantly difference at p-value < 0.05 (t-test).

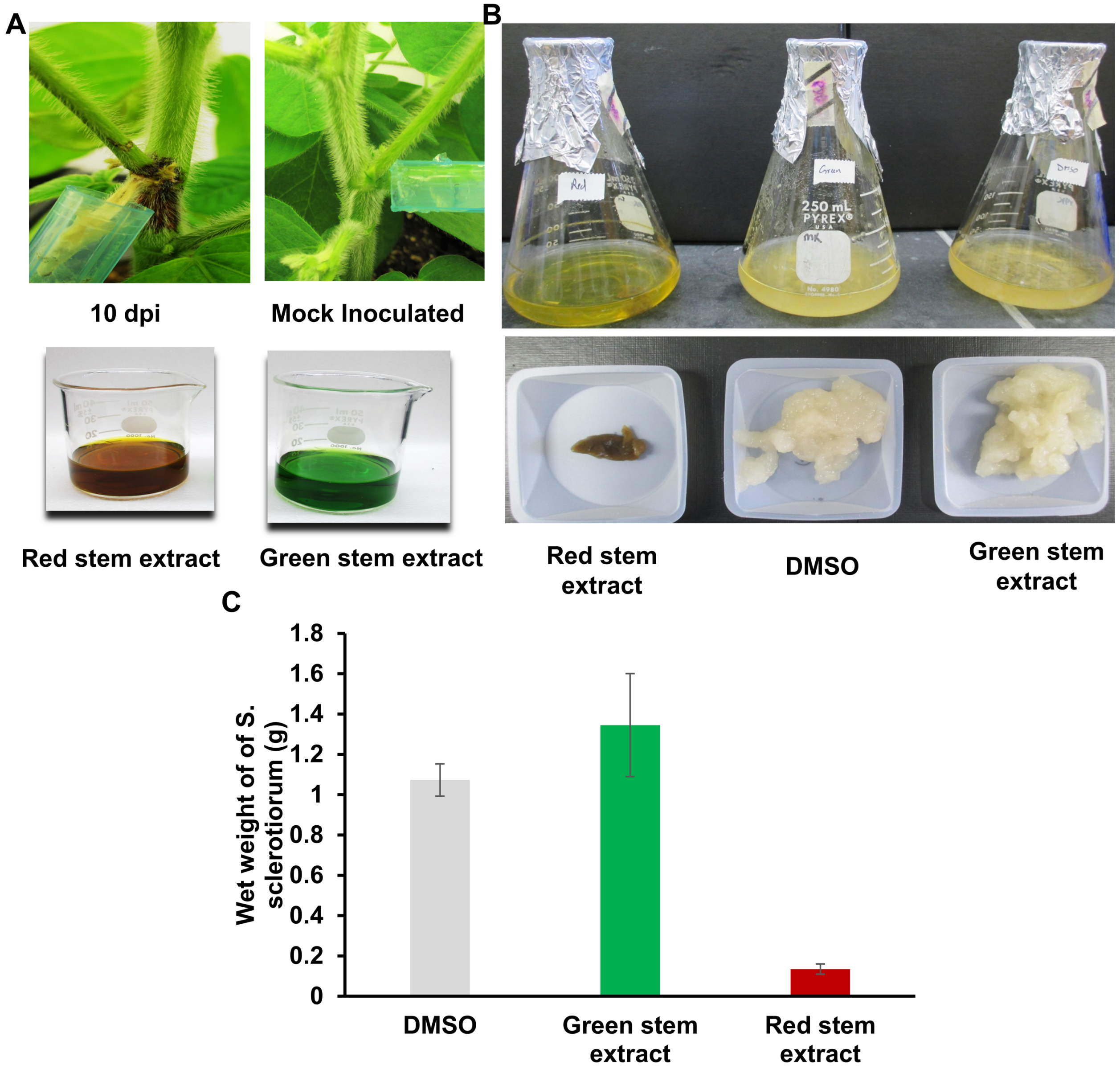

Supplementary Figure 7. (A) Red and green stem extract of a R line plant infected with *S. sclerotiorum* and a S line plant mock inoculated. Extraction was performed 10 dpi, (B) Fungal biomass after growth in PDB cultures containing the red stem extract, DMSO, or green stem extract, (C) weight of fungal biomass in PDB cultures containing the red stem extract, green stem extract, or DMSO.
